# Transcriptome comparisons of in vitro intestinal epithelia grown under static and microfluidic gut-on-chip conditions with in vivo human epithelia

**DOI:** 10.1038/s41598-021-82853-6

**Published:** 2021-02-05

**Authors:** Kornphimol Kulthong, Guido J. E. J. Hooiveld, Loes Duivenvoorde, Ignacio Miro Estruch, Victor Marin, Meike van der Zande, Hans Bouwmeester

**Affiliations:** 1 Division of Toxicology, Wageningen University, P.O. box 8000, 6700 EA Wageningen, The Netherlands; 2 Wageningen Food Safety Research, P.O. Box 230, 6700 AE Wageningen, The Netherlands; 3 National Nanotechnology Center (NANOTEC), National Science and Technology Development Agency, Pathum Thani, 12120 Thailand; 4 Nutrition, Metabolism and Genomics group, Division of Human Nutrition and Health, Wageningen University, Wageningen, The Netherlands

**Keywords:** Gastrointestinal models, Lab-on-a-chip

## Abstract

Gut-on-chip devices enable exposure of cells to a continuous flow of culture medium, inducing shear stresses and could thus better recapitulate the in vivo human intestinal environment in an in vitro epithelial model compared to static culture methods. We aimed to study if dynamic culture conditions affect the gene expression of Caco-2 cells cultured statically or dynamically in a gut-on-chip device and how these gene expression patterns compared to that of intestinal segments in vivo. For this we applied whole genome transcriptomics. Dynamic culture conditions led to a total of 5927 differentially expressed genes (3280 upregulated and 2647 downregulated genes) compared to static culture conditions. Gene set enrichment analysis revealed upregulated pathways associated with the immune system, signal transduction and cell growth and death, and downregulated pathways associated with drug metabolism, compound digestion and absorption under dynamic culture conditions. Comparison of the in vitro gene expression data with transcriptome profiles of human in vivo duodenum, jejunum, ileum and colon tissue samples showed similarities in gene expression profiles with intestinal segments. It is concluded that both the static and the dynamic gut-on-chip model are suitable to study human intestinal epithelial responses as an alternative for animal models.

## Introduction

Current toxicological safety studies of chemicals and pharmaceuticals often rely on the use of laboratory animals. The use of animals not only is time consuming, considered unethical and expensive, but importantly also raises scientific questions on the differences in physiology of laboratory animals compared to humans^1–3^. To refine, reduce or ultimately replace the use of animal models (the 3R principle)^4^, in vitro assays have been intensively studied^5–7^. By using human cells, specific functions of the organ of origin can be emulated in vitro. Here, we focused on human intestinal cells that are extensively used to model the human intestinal epithelium.

With the emergence of microfluidic technology several organ-on chip platforms have been launched^8,9^. More specifically, gut-on-chip devices have been introduced that allow to culture epithelial cells under continuous perfusion and physiological shear stress attempting to better recapitulate the environment in the human intestine compared to static culture methods^10–13^. While stem cell-based intestinal models can be used to study the human intestinal function, the reproducibility and culture efficiency of the models in vitro is still challenging^14,15^. Therefore, human intestinal epithelial cell line-based models, specifically Caco-2 cells, currently remain the most widely used and accepted in vitro model for toxicological safety studies^16–18^. Despite the variety in existing gut-on-chip models, there are only a few studies that evaluated the basal gene expression of Caco-2 cells compared to that of human intestinal (in vivo) tissues^19^. Kim et al. published such a comparison of a specific subclone of Caco-2 cells, but due to the very limited sample size in this study the results have to be interpreted with caution. In addition, the consequences of different chip designs need to be interpreted with care, as the resulting differences in shear stress induce different phenotypical and functional changes^20^. To the best of our knowledge, no other comparative studies addressing this issue in the commonly used wild type Caco-2 cell line have been published so far.

The aim of the current study was to comprehensively investigate the effects of dynamic flow conditions on the gene expression profile and affected biological pathways of Caco-2 cells compared to the gene expression profile of Caco-2 cells cultured under static conditions. Next, the gene expression profiles of Caco-2 cells, cultured under both conditions, were compared with those of healthy human in vivo intestinal tissues. For this, we retrieved data from publicly available gene expression databases. Briefly, Caco-2 cells were grown for 21 days in Transwells according to a standard protocol^21^, and in our gut-on-chip device^12,13^. Gene expression data were obtained using a microarray platform and differential expression was determined by a bioinformatics approach. Linear models and an intensity-based moderated t-statistic were used for identification of differentially expressed genes and gene set enrichment analysis (GSEA) for identification of affected biological pathways. The differential expression of intestine-specific genes in Caco-2 cells was compared to those reported for different regions of human intestinal tissues in vivo^22^.

## Results

### Cellular morphological assessment

**Figure 1:**
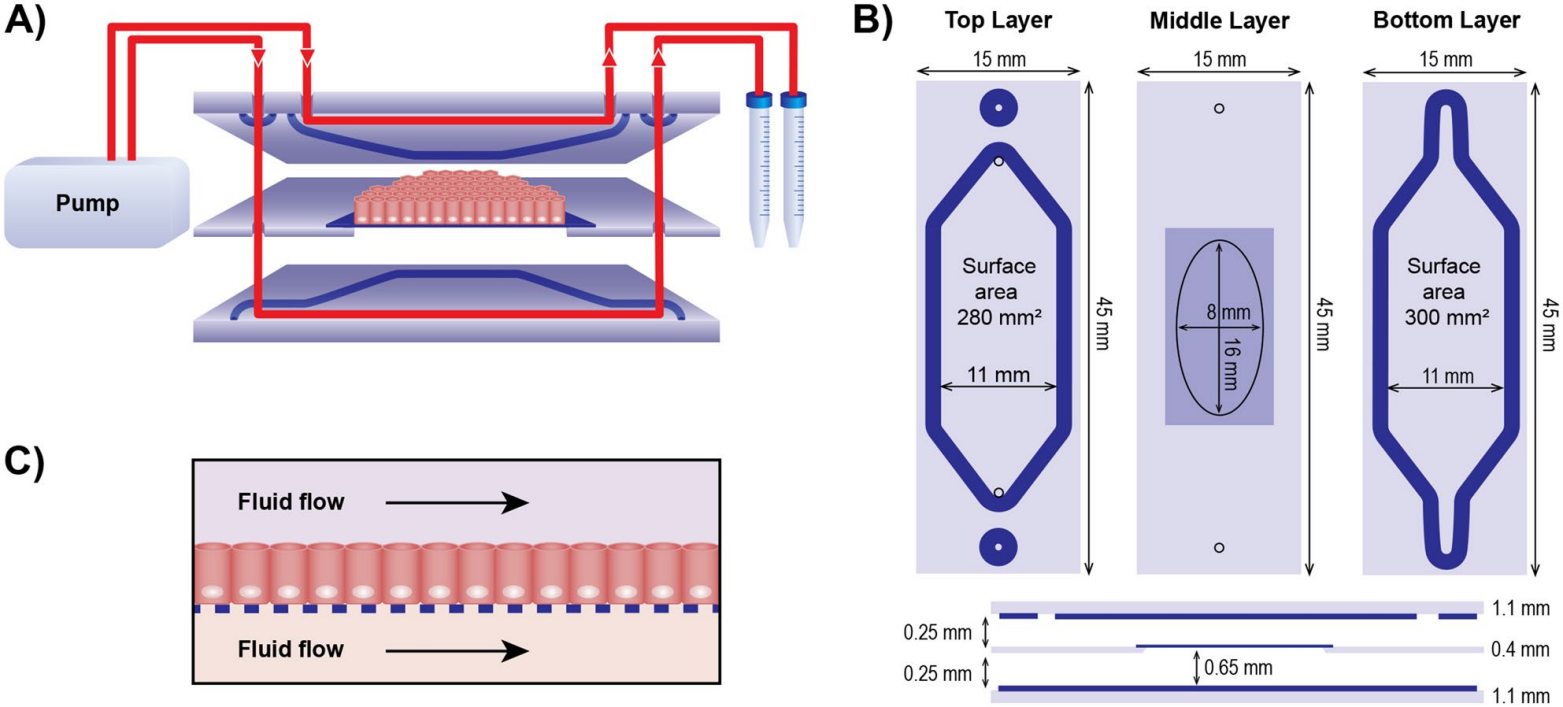
Schematic illustration of the experimental setup of the gut-on-chip (**A**) Schematic design of the microfluidic system showing a vertical cross-section of the glass chip. The red lines represent the flows in and out of both compartments. (**B**) Schematic drawing of the three glass layers that form the chip, the oval in the middle layer represents a cut-out in the glass and the darker square represents the porous membrane covering the cut-out. The dark blue lines in (**A**) and (**B**) represent the gaskets that form the boundaries of the compartments when the three glass layers are pressed together in the chip holder. (**C**) Schematic drawing showing how cells were exposed to fluid flow.

**Figure 2:**
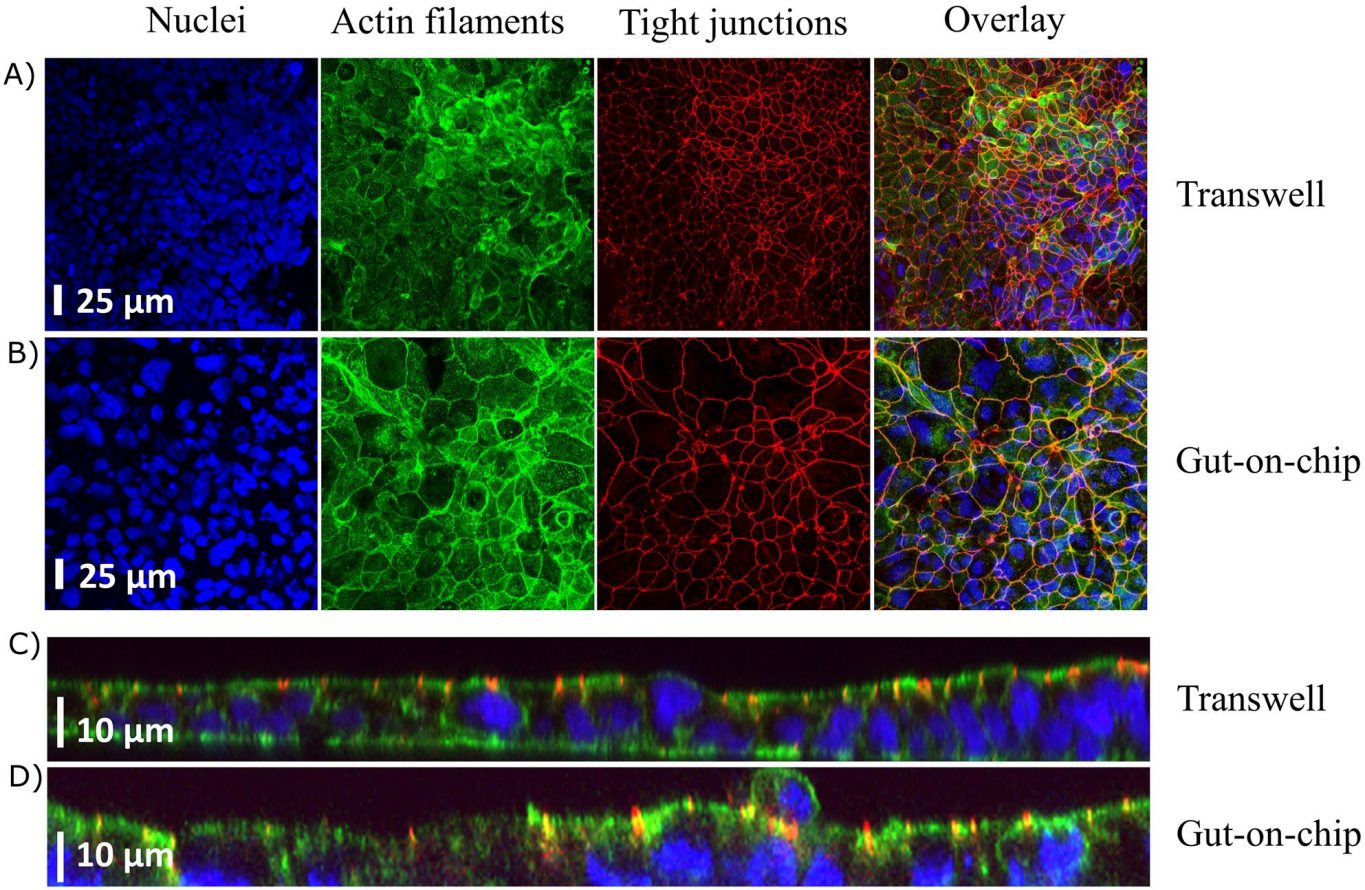
Cell morphology of Caco-2 cells after 21 days of culture under static (Transwell) and dynamic, with a flow of 100 µL/h, (gut-on-chip) conditions, visualized by confocal microscopy. Top views of the cell layer in (**A**) the Transwell and in (**B**) the gut-on-chip. Vertical cross-sections of the cell monolayer in (**C**) the Transwell and (**D**) the gut-on-chip. Actin filaments (Phalloidin) in green, cell nuclei (DAPI) in blue, and tight junctions (ZO-1/TJP1) in red.

Monolayer integrity of Caco-2 cells grown for 21 days in the gut-on-chip under dynamic flow (Fig. 1A–C) or in the static Transwell was assessed using confocal microscopy imaging. The top views of representative images are shown in Fig. 2A,B. Caco-2 cells grown under continuous flow showed a comparable monolayer formation and cell morphology at day 21 to cells grown under static conditions, as reflected by immunofluorescence staining of nuclei (blue), actin filaments (green) and tight junctions (red). Cells cultured under flow, however, seemed to be larger than those grown under static conditions. Vertical cross-sections of the monolayers, created by Z-stacks (Fig. 2C,D), showed cell polarization with core bundles of actin filaments in the microvilli and tight junctions on the apical side, in cells grown under both conditions. The cell heights were comparable in both systems, reaching ~ 10 µm at day 21.

### Gene expression in Caco-2 human epithelial cells under static and dynamic conditions

Genome-wide changes in gene expression in Caco-2 cells grown under dynamic culture conditions in the gut-on-chip were identified by comparison of gene expression of cells grown under static versus dynamic culture conditions. After 21 days of culturing, total RNA was isolated and gene expression was analyzed using Affymetrix GeneChips.

**Figure 3:**
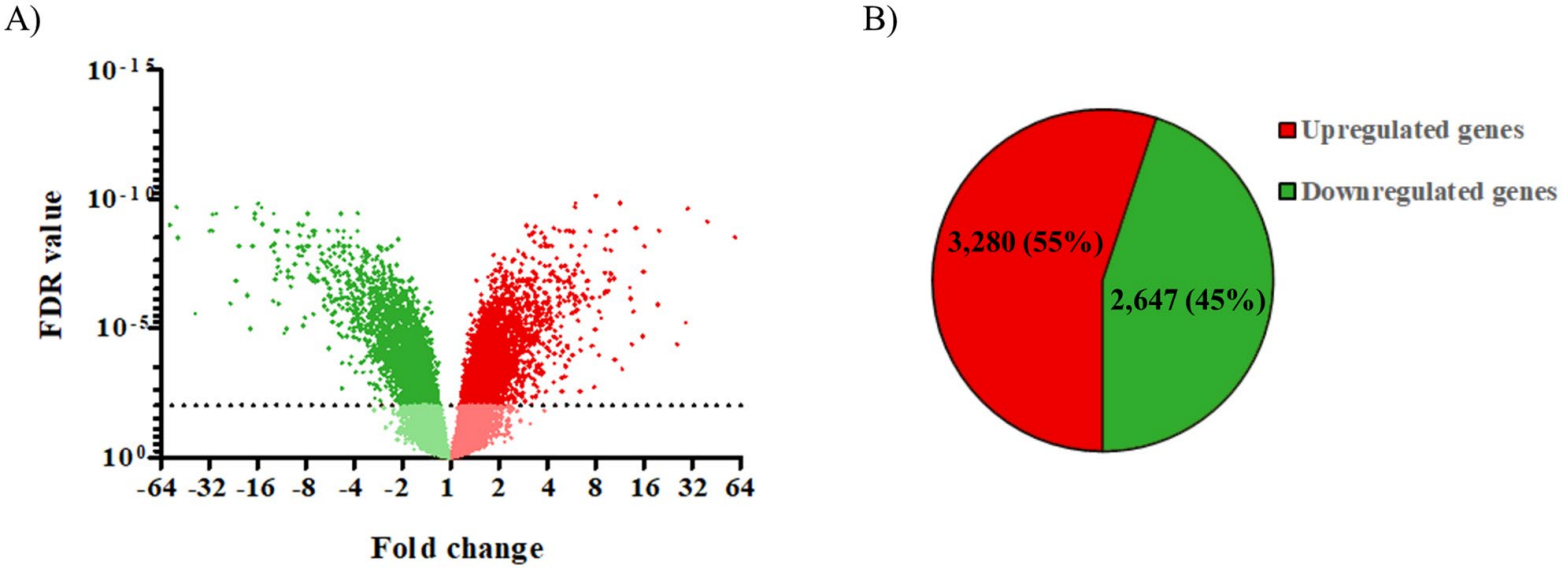
Overview of the differential gene expression in Caco-2 cells grown in a gut-on-chip device versus Transwell inserts after 21 days of culture. (**A**) Volcano plot of all differentially expressed genes where the FDR value of each gene is plotted against the fold change in expression; the dotted line indicates an FDR of 0.01. (**B**) The number and percentages of differentially expressed genes in Caco-2 cells grown in a gut-on-chip device compared to cells grown in Transwell inserts.

After data processing, differential gene expression was visualized in a volcano plot (Fig. 3A). The expression of 29,635 genes in Caco-2 cells grown in the gut-on-chip device was compared with that in cells grown under static conditions. In total, 5927 differentially expressed genes were observed in the gut on chip (3280 upregulated and 2647 downregulated) with a FDR < 0.01 (Fig. 3B). The top 10 most up- and downregulated genes in cells grown in the gut-on-chip device, compared to cells grown in the Transwell inserts, are listed in Table 1. Compared to the Transwell inserts, the most upregulated gene in cells grown in the gut-on-chip device was metallothionein 1H (*MT1H*; log_2_FC = 5.89) coding for metallothionein 1H protein, whereas the gene glucose-6-phosphatase catalytic subunit (*G6PC*; log_2_FC = -6.79) coding for glucose-6-phosphatase catalytic subunit protein was most downregulated.

**Table 1:** Top 10 most up- and downregulated genes in Caco-2 cells cultured under dynamic conditions compared to static conditions after 21 days of culture.

EntrezID	Gene symbol	Gene full name	Core function in cells^a^	Log_2_ FC	P-value	FDR
**Upregulated genes**						
4496	MT1H	Metallothionein 1H	Mineral absorption, Metal binding	5.89	5.6E−12	3.0E−09
4495	MT1G	Metallothionein 1G	Mineral absorption, Metal binding	5.32	5.0E−13	7.4E−10
301	ANXA1	Annexin A1	Anti-inflammation	4.91	8.0E−14	2.4E−10
54658	UGT1A1	UDP glucuronosyltransferase family 1 member A1	Metabolism	4.87	2.1E−07	6.1E−06
10202	DHRS2	Dehydrogenase/reductase 2	Metabolism	4.68	2.3E−06	4.0E−05
4501	MT1X	Metallothionein 1X	Mineral absorption, Metal binding	4.31	1.9E−12	1.7E−09
10410	IFITM3	Interferon induced transmembrane protein 3	Immune system	4.30	2.5E−08	1.2E−06
9120	SLC16A6	Solute carrier family 16 member 6	Membrane transporter	4.02	1.5E−11	6.7E−09
7348	UPK1B	Uroplakin 1B	Cellular development and growth	3.99	4.3E−10	6.7E−08
3429	IFI27	Interferon alpha inducible protein 27	Immune system	3.98	9.9E−07	2.0E−05
**Downregulated genes**						
2538	G6PC	Glucose-6-phosphatase catalytic subunit	Metabolism	− 6.79	4.7E−13	7.3E−10
229	ALDOB	Aldolase, fructose-bisphosphate B	Metabolism	− 5.81	8.3E−13	1.0E−09
284099	C17orf78	Chromosome 17 open reading frame 78	Unknown	− 5.67	4.3E−14	2.1E−10
56624	ASAH2	N-acylsphingosine amidohydrolase 2	Metabolism, Biosynthesis	− 5.64	6.1E−12	3.1E−09
6476	SI	Sucrase-isomaltase	Metabolism, Digestion	− 5.28	7.4E−08	2.7E−06
795	S100G	S100 calcium binding protein G	Mineral absorption, Membrane transporter	− 4.99	2.2E−12	1.7E−09
1557	CYP2C19	Cytochrome P450 family 2 subfamily C member 19	Metabolism	− 4.94	2.0E−12	1.7E−09
4547	MTTP	Microsomal triglyceride transfer protein	Lipid digestion and absorption	− 4.92	2.2E−13	3.9E−10
79853	TM4SF20	Transmembrane 4 L six family member 20	Cell growth, proliferation and activities	− 4.84	1.7E−13	3.6E−10
	NRN1	Interferon alpha inducible protein 27	Neurite outgrowth	− 4.55	2.1E−08	1.1E−06

^a^References on the gene functions are provided in Supplementary Table S3.

### Overview of gene set enrichment analysis

**Figure 4:**
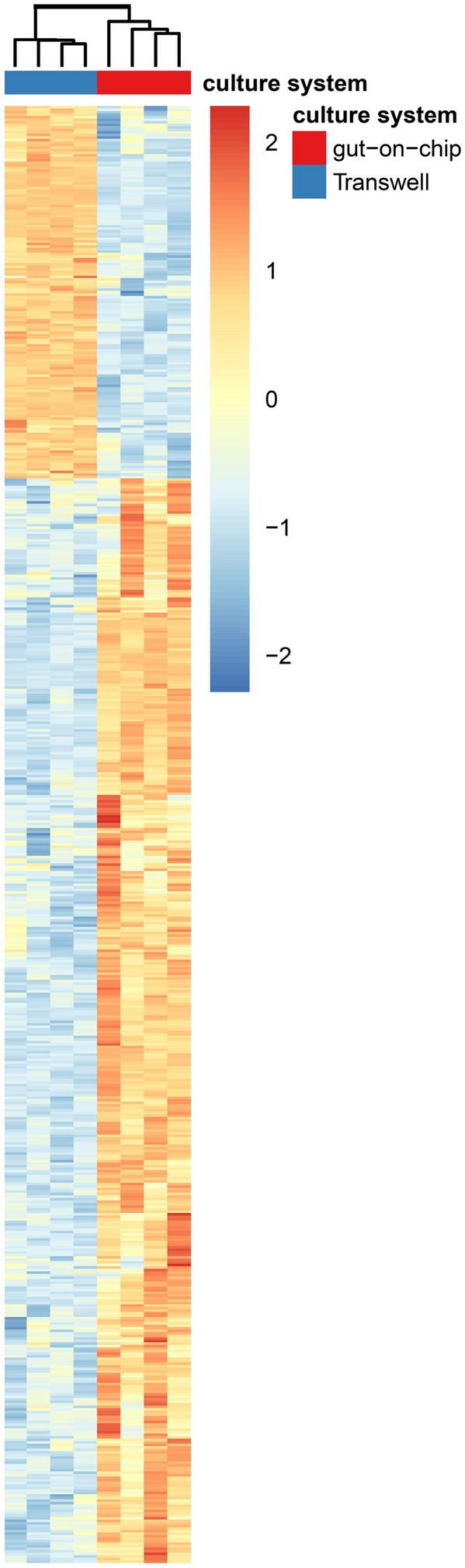
Heatmap showing the leading edge genes (575 genes in total) of 24 enriched gene sets. In the GSEA, gene expression patterns of Caco-2 cells cultured in a gut-on-chip were compared with those of Caco-2 cells cultured in Transwells. Heatmaps of selected genes were made using the R library pheatmap (version 3.6.3, https://cran.r-project.org/web/packages/Pheatmap/index.html)^23^.

GSEA was performed to elucidate whether biological processes were potentially affected in cells cultured under dynamic conditions compared to cells cultured under static conditions, based on gene expression data. The studied pathways were derived from the KEGG database. This database is structured into KEGG categories that are subdivided into category subgroups and each category subgroup contains various pathways, each represented by a gene set. As described in the material section we have considered gene sets belonging to 5 categories namely ‘metabolism’, ‘genetic information processing’, ‘environmental information processing’, ‘cellular processes’ and ‘organismal systems’ (BRITE Functional Hierarchy level 1). This resulted in the analysis of 225 gene sets. Of these 225 gene sets, 108 gene sets were differently expressed, of which 52 gene sets were upregulated in Caco-2 cells cultured in the gut-on-chip versus Caco-2 cells cultured in Transwells and 56 gene sets were downregulated in Caco-2 cells grown in the gut-on-chip (p-value < 0.05 and FDR < 0.25). The most prominently upregulated gene set in Caco-2 cells cultured in the gut-on-chip represented the ‘ribosome biogenesis’ pathway (normalized enrichment score, NES = 2.52) under the KEGG category ‘genetic information processing’ and KEGG category subgroup ‘translation’ (suppl. Table S1). The most prominently downregulated pathway in Caco-2 cells cultured under dynamic conditions represented the ‘protein digestion and absorption’ pathway (NES = − 2.23) under the KEGG category ‘organismal system’ and KEGG category subgroup ‘digestive system’ (suppl. Table S2). The gene expression analysis was continued by focusing on up- and downregulated gene sets that represent pathways belonging to crucial small intestinal functions, core signaling and cell survival. Twenty-four gene sets, belonging to the KEGG category subgroups: ‘xenobiotics biodegradation and metabolism’, ‘membrane transport’, ‘cellular transport’, ‘immune system’, ‘signal transduction’, ‘cell growth and death’ and ‘digestive system’ (Table 2), were evaluated. Various gene sets in the KEGG category subgroups ‘xenobiotics biodegradation and metabolism’ and ‘digestive system’ were downregulated. Various gene sets in the KEGG category subgroups ‘cellular transport’, ‘immune system’ and ‘cell growth and death’ were upregulated, In the 24 enriched gene sets, there were 575 genes that were contributing most to the enrichment, the so called leading edge genes, which are shown in a heatmap in Fig. 4.

**Table 2:** Most enriched gene sets representing pathways associated with intestinal cell function, core signaling and cell survival in Caco-2 cells cultured under dynamic conditions compared to static conditions.

KEGG pathway name	KEGG category	KEGG category subgroup	Size	NES	p-value	FDR
**Upregulated gene sets**						
Endocytosis	Cellular processes	Transport and catabolism	243	1.42	3.4E−03	7.8E−02
NOD-like receptor signaling pathway	Organismal system	Immune system	168	1.93	0.00E+00	5.6E−04
RIG-I-like receptor signaling pathway	Organismal system	Immune system	67	1.60	3.8E−03	2.8E−02
Cytosolic DNA-sensing pathway	Organismal system	Immune system	58	2.01	0.0E+00	1.7E−04
IL-17 signaling pathway	Organismal system	Immune system	93	1.72	3.7E−04	9.5E−03
MAPK signaling pathway	Environmental information processing	Signal transduction	292	1.60	0.0E+00	2.7E−02
TGF-beta signaling pathway	Environmental information processing	Signal transduction	92	1.47	1.3E−02	5.9E−02
Jak-STAT signaling pathway	Environmental information processing	Signal transduction	158	1.41	1.1E−02	8.3E−02
NF-kappa B signaling pathway	Environmental information processing	Signal transduction	97	1.96	0.0E+00	3.5E−04
TNF signaling pathway	Environmental information processing	Signal transduction	110	2.00	0.0E+00	2.2E−04
Cell cycle	Cellular processes	Cell growth and death	124	1.92	0.0E+00	6.5E−04
Apoptosis	Cellular processes	Cell growth and death	133	1.66	1.8E−04	1.6E−02
Necroptosis	Cellular processes	Cell growth and death	128	1.77	0.0E+00	6.2E−03
p53 signaling pathway	Cellular processes	Cell growth and death	72	1.70	1.1E−03	1.1E−02
Cellular senescence	Cellular processes	Cell growth and death	158	1.39	1.4E−02	9.3E−02
**Downregulated gene sets**						
Drug metabolism-cytochrome P450	Metabolism	Xenobiotics biodegradation and metabolism	62	− 2.00	0.0E+00	4.4E−04
Drug metabolism-other enzymes	Metabolism	Xenobiotics biodegradation and metabolism	70	− 1.42	2.5E−02	8.5E−02
ABC transporters	Environmental information processing	Membrane transport	45	− 1.48	2.9E−02	5.8E−02
Carbohydrate digestion and absorption	Organismal system	Digestive system	39	− 2.19	0.0E+00	5.7E−05
Protein digestion and absorption	Organismal system	Digestive system	83	− 2.23	0.0E+00	1.1E−04
Fat digestion and absorption	Organismal system	Digestive system	41	− 2.17	0.0E+00	3.8E−05
Vitamin digestion and absorption	Organismal system	Digestive system	23	− 2.00	0.0E+00	4.8E−04
Phosphatidylinositol signaling pathway	Environmental information processing	Signal transduction	99	− 1.60	2.2E−03	2.9E−02
AMPK signaling pathway	Environmental information processing	Signal transduction	119	− 1.39	2.1E−02	9.9E−02

### Transcriptomic comparison of Caco-2 cells grown in the gut-on-chips and transwells, and human in vivo data

**Figure 5:**
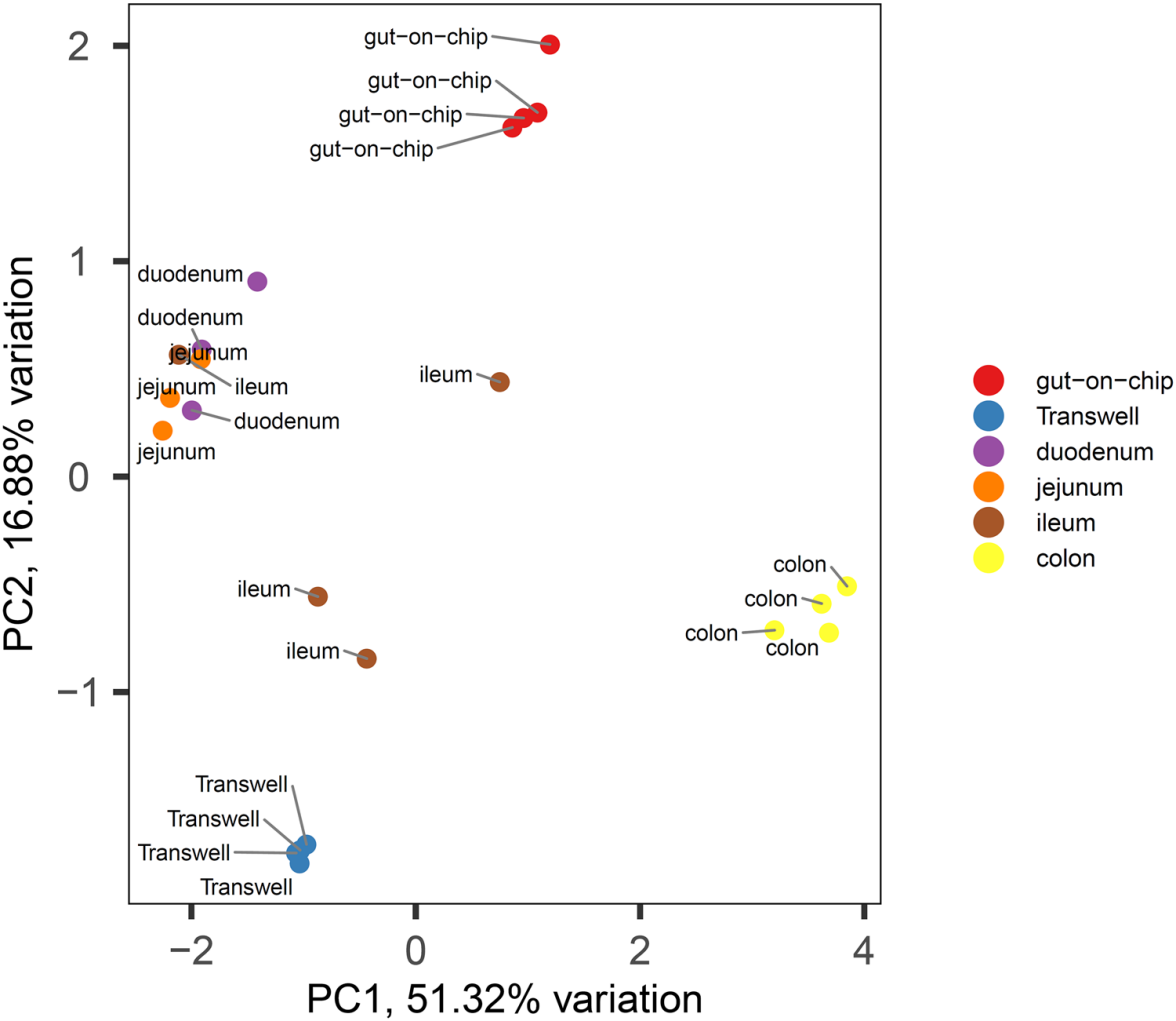
PCA plot of genomic expression data of 483 of human intestine-specific genes^24^ from human duodenum, jejunum, ileum and colon tissues, and Caco-2 cells cultured in a gut-on-chip or Transwell system.

**Figure 6:**
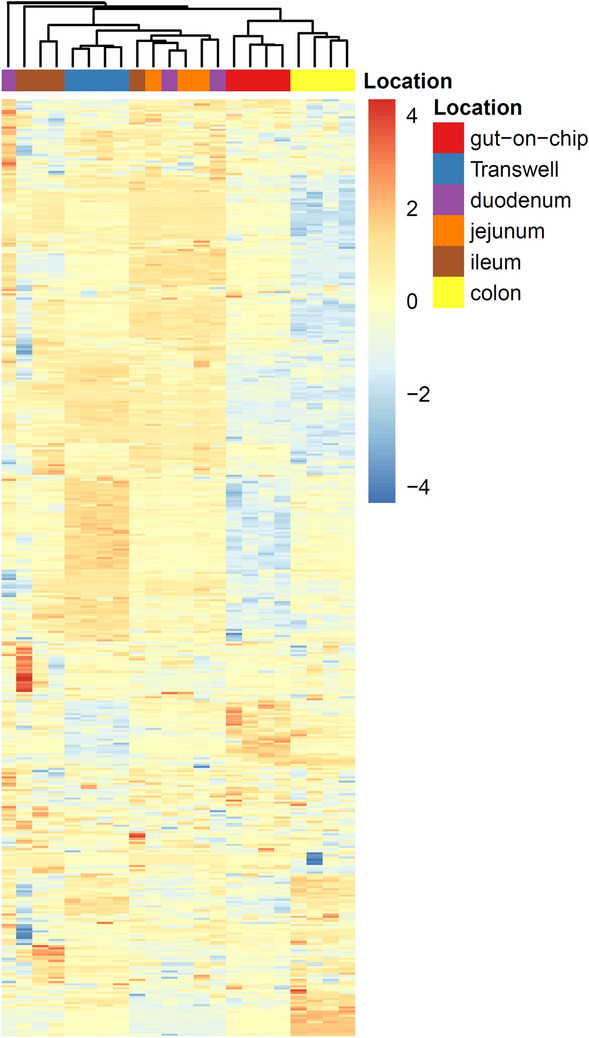
Hierarchical clustering of the expression data of 483 of human intestine-specific genes in Caco-2 cells grown in the gut-on-chip or Transwell and in human intestinal tissue samples. Heatmaps of selected genes were made using the R library pheatmap (version 3.6.3, https://cran.r-project.org/web/packages/Pheatmap/index.html)^23^.

Next, we compared the gene expression profiles of the Caco-2 cells grown under dynamic and static conditions in vitro, with human intestinal in vivo gene expression profiles. For this, we selected a publicly available gene expression data set from the human proteome atlas that contained data of human intestinal tissues^24^. The gene expression profiles were evaluated by a principal component analysis (PCA). A PCA scatterplot representing the first two principal components based on the transcriptome profiles from 14 human in vivo samples and 4 samples each of the Transwell and the gut-on-chip cell culturing system is shown in Fig. 5. PC1 and PC2 explain 51.32% and 16.88% of the total variation, respectively. Samples from the cells cultured in the gut-on-chip device and in the Transwells clustered together showing the low variation and high robustness in each in vitro data set. This was also observed for the in vivo colon samples, while the small intestinal samples (especially the ilieum samples) clustered somewhat more scattered. The first component (PC1) indicates that Caco-2 cells cultured in gut-on-chip clusters were more distant from the clusters of jejunum and duodenum samples, and closer to the colon in vivo samples than the Caco-2 cells cultured in the Transwell system. The second component (PC2) indicates that the in vivo data sets located between the two clusters of the in vitro samples (i.e. gut-on-chip and Transwell). In the database of the human proteome atlas, from which we selected the intestinal tissue in vivo data sets, 764 genes have been annotated as intestine specific, 483 (63%) of these genes were expressed in our gene expression data from Caco-2 cells cultured under static or dynamic conditions and data from selected tissue samples from human duodenum, jejunum, ileum and colon^22^ and were hierarchically clustered (Fig. 6). The clustering pattern of the various in vitro and in vivo samples as observed by PCA is confirmed by the hierarchical clustering based on the intestine specific 483 genes.

## Discussion

In this study we provide a comprehensive overview on whole genome differential gene expression in Caco-2 cells when cultured under dynamic in vitro culture conditions versus static in vitro culture conditions. In addition, we compared the transcriptome profiles of our in vitro experiments with the transcriptome profiles as observed in human (in vivo) intestinal segments. Monolayers of Caco-2 cells grown in conventional static systems have been widely used to study effects of exposure to chemicals to predict the in vivo human intestinal epithelial responses^25–27^. However, in vivo the epithelial cells of the intestinal wall experience physical forces including strain, fluid shear stress, and villous motility. Shear stresses to cells might be important triggers in the development and maturation of epithelial cells^28^. We here show a differential expression of 5927 genes in Caco-2 cells induced by dynamic culture conditions as compared to static culture conditions. The shear stress of ~ 0.002 dyne/cm^2^ in our model induced comparable changes in gene expression profiles as reported before in a model that exposed Caco-2 cells to an estimated shear stress of ~ 0.02 dyne/cm^2^^19^. No other studies on the effects of shear forces on Caco-2 cells based on transcriptomics data could be found. As reported previously, cells grown under dynamic conditions seemed to be larger compared to cells grown under static conditions^12,13^. The morphology of Caco-2 cells has been shown to be affected by differences in shear forces. Using a microfluidic device with decreasing dimensions, thus increasing shear forces, Delon et al. have studied the consequences of increased shear forces on cell morphology and functionality of 5 day old Caco-2 cells. The authors reported that increasing shear forces resulted in increased cell heights, microvilli formation and mucus production by Caco-2 cells^20^, and corroborate our findings on cell morphology at low shear forces. Interestingly, for two other types of cells the effects of shear forces on gene expression have been studied in detail, namely for human vascular endothelial cells (with fluid shear stresses ranging from 1.5 to 15 dyne/cm^2^;^29,30^) and on murine proximal tubular epithelial cells (with fluid shear stresses ranging from 0 to 1.9 dyne/cm^2^;^31^). These studies revealed clear effects of fluid shear stresses on gene expression profiles in the cells. A comparison of the findings on affected genes and processes in these studies with our results will be discussed further below.

At the individual gene level, fluid flow applied to Caco-2 cells resulted in the upregulation of several genes related to mineral absorption/metal binding. Highly upregulated genes were the metallothionein genes (i.e. MT1H, MT1G, MT1X) that provide protection against metal toxicity^32^ and oxidative stress^33^. Interestingly, the modulation of metallothionein genes has been observed in endothelial cells in vitro upon physical stress^30,34^.

The KEGG category ‘xenobiotics biodegration and metabolism’ was down regulated under dynamic conditions. Various individual genes related to cellular metabolism (i.e. *G6PC, ALDOB, ASAH2*) were downregulated under dynamic conditions. Exceptions, however, were genes coding for *UGT1A1* and *CYP1A1* that were extremely upregulated in Caco-2 cells cultured under dynamic conditions (top 20 most upregulated genes). The latter genes relate to isoforms of enzymes that are important in drug and xenobiotic metabolism in the small intestine. *UGT1A1* catalyzes glucuronic acid conjugation to a nucleophilic substrate^35,36^ and *CYP1A1* is involved in the modification of aromatic hydrocarbons. Gene expression of *UGT1A1* and *CYP1A1* is regulated by the aryl hydrocarbon receptor (AhR)^37,38^. The AhR gene and AhR dependent genes (i.e. *CYP1B1, TIPARP, PTGS2*) were also upregulated under dynamic culturing conditions. The upregulated expression of this functional group of AhR regulated genes has also been observed in human endothelial cells exposed to shear stress^39–41^.

We next set off to analyze if the differential gene expression also affected biological pathways using GSEA. The most relevant affected pathways for intestinal functions and core signaling pathways were listed in Table 2. It is of interest that gene sets involved in inflammatory pathways (i.e. IL-17 signaling pathway, cytosolic DNA-sensing pathway) were upregulated in Caco-2 cells that were cultured in the gut-on-chip. This included the upregulation of genes for the NOD-like receptor, RIG-I receptor signaling pathways that are involved in the innate immune responses^42,43^. This indicates that fluid shear stresses might modulate the defense mechanism of intestinal epithelial cells by stimulating the innate immune response. Miravete et al. observed that human proximal tubular cells (HK-2) exposed to a shear stress of 0.1 dyne/cm^2^ activated the differentiation of monocytes into macrophages by secretion of TNF-alpha^44^, which also are elements of the innate immune system.

Various signaling pathways (e.g. MAPK, TGF-beta, Jak-STAT, NF kappa B, TNF, p53) belonging to the ‘signal transduction’ and ‘cell growth and death’ KEGG category subgroups were upregulated in Caco-2 cells grown under dynamic conditions. These pathways have important regulatory roles in a wide variety of cellular processes including cell proliferation, differentiation, apoptosis and stress responses in mammalian cells^45–49^. While the effects of shear stresses on signaling processes in intestinal cells is poorly studied, much more is known from endothelial cells and these findings corroborate the results observed in the present study. In endothelial cells, shear stress-induced IL-8 gene expression (4.2 dyne/cm^2^) regulated by MAPK signaling^50^. TGF-beta signaling is also described to be induced in endothelial cells by shear stress of 10 dyne/cm^2^^51^. NF kappa B signaling, stimulating pro-inflammatory cytokine and chemokine release, was activated by a shear stress of 15 dyne/cm^2^ in endothelial cells^52^.

Compound metabolism pathways, drug metabolism-cytochrome P450 and other enzymes, belonging to the ‘xenobiotics biodegradation and metabolism’ KEGG category subgroup were downregulated in Caco-2 cells exposed to shear stress, as was also observed at the individual gene expression level with the exception of *UGT1A1* and *CYP1A1*(AhR dependent genes). Studies with a different subclone of Caco-2 cells (i.e. Caco-2BBE) cultured under a shear stress of 0.02 dyne/cm^2^^11^, or under a range of shear stresses (ranging from 0.002 to 0.03 dyne/cm^2^^20^ showed a shear stress dependent increase in activity of the drug metabolizing cytochrome CYP3A4 enzyme compared to cells cultured under static conditions^11,20^. In our results, the pathways associated to general cellular metabolism were also downregulated (suppl. Table S2). This is in line with a study in renal epithelial cells where a downregulation of gene expression at several levels for cellular homeostasis, including fatty acid, amino acid and cholesterol metabolism, was observed after exposure to a shear stress of 1.9 dyne/cm^2^^31^. Nutrient digestion and absorption by epithelial cells might also be affected by fluid flow exposure as indicated by the downregulation of gene sets associated with those processes (i.e. gene sets for the protein, carbohydrate and fat digestion and absorption pathways belonging to the ‘digestive system’ KEGG category subgroup). This has also been observed in endothelial cells, in which shear stresses (20 dyne/cm^2^) reduced the expression of genes involved in glucose absorption^53^.

Lastly, we compared the gene expression patterns of both our in vitro models with those of samples taken from different intestinal segments as reported in literature^22^. In a PCA analysis of all data samples from our in vitro models cluster together in two separate groups that both are different from the in vivo gene expression clusters. The PCA clustering revealed that the gene expression profiles of Caco-2 cells cultured under both culture conditions more closely recapitulated small intestine gene expression than the colonic gene expression. However, the profiles of the Caco-2 cells grown under shear stress were clustered more towards the colonic samples than the Caco-2 cells grown under static conditions (Fig. 5). Interestingly, the duodenal and jejunum samples clustered together, while the gene expression of the ileum samples (from the same donors) seemed to be much more variable. To the authors knowledge, there is only one other study reporting on the transcriptomes of Caco-2 cells cultured in gut-on-chip and Transwell devices compared with in vivo data^19^. However, with the very limited number of samples (n = 2) the authors included it is quite challenging to draw the strong conclusion from this study.

In conclusion, our study provides a comprehensive profile of altered gene expression in Caco-2 cells under flow culturing conditions versus culturing under static conditions. The responses were mainly related to cellular homeostasis, immunological responses, cell growth and dead, as well as signal transduction. While general cellular metabolism and absorption pathways were repressed, specific genes in xenobiotic biotransformation pathways were induced upon exposure to fluid flow. Interestingly, comparable responses have been noted in endothelial and renal tubular epithelial cells that were also exposed to shear stress. Our unbiased comparison with global gene expression in samples from intestinal segments did not reveal a striking similarity with any of these segments. The results obtained do not apparently favor one of the two in vitro models and it can be concluded that both model systems can be equally well used to study human intestinal epithelial responses, thus selection may depend on the endpoint of interest. For instance, to derive uptake rates for pharmacokinetic modelling the robust and routinely used Transwell models might be the preferred approach^54^, while to emulate complex interactions in the intestine organ-on-chip models might be the preferred model^55–57^. It should be kept in mind that some specific gene functions are differently modulated in each model. This information may be used to further advance the applicability of flow conditions in in vitro cells systems for use as alternatives for animal models.

## Materials and methods

### Chemicals and reagents

Dulbecco’s Modified Eagle Medium (DMEM), Hank’s balanced salt solution (HBSS), penicillin/streptomycin and Bovine serum albumin (BSA) were purchased from Sigma-Aldrich (Zwijndrecht, The Netherlands). Fetal bovine serum (heat inactivated) (FBS), MEM-Non-essential amino acids Phosphate Buffered Saline (PBS) were obtained from Fisher Scientific (Landsmeer, The Netherlands).

### Design of the gut-on-chip system

The microfluidic gut-on-chip device has been developed and described previously^12,13^. Briefly, the chip consists of three 15 × 45 mm (width x length) re-sealable glass slides that result in two flow chambers (i.e. an upper apical (AP) and lower basolateral (BL) chamber) upon assembly (see Fig. 1A; Micronit, Enschede, The Netherlands). Both the upper and lower glass slides were spaced from the middle layer membrane by a 0.25 mm thick silicone gasket. The flow chambers were separated by a glass slide containing a polyester (PET) porous cell culture membrane with a 0.4 µm pore size and a cell culture area of ~ 1.6 cm^2^. The volume of the AP chamber is 75 mm^3^ with a chamber height of 0.25 mm (membrane to top layer) and the BL chamber is 110 mm^3^ with a chamber height of 0.65 mm (bottom layer to membrane), resulting in a total volume of 185 mm^3^ (µL) of the device (Fig. 1B). The chip was placed in a chip holder with a quick locking mechanism, constructed for connection of external capillaries to the chip via specific ferrules to ensure tight connections and a leak-free system.

The constant flow was introduced to the chip using a microsyringe pump (NE-4000, New Era Pump Systems, Inc.) equipped with two polypropylene syringes (30 mL, Luer-locktm, Becton, Dickinson and company), with each syringe connected to either the AP or the BL compartment using Ethylene Propylene (FEP) tubing (0.50 mm inner diameter, with a length of 25 cm and 10 cm for the inlet and outlet, respectively). Before the start of each experiment, all tubing and chips were sterilized using an autoclave and rinsed with 70% ethanol. Tubing and chips were prefilled with medium to eliminate air bubbles in the system. The entire system was put in an incubator at 37 °C to maintain cell culture conditions.

### Cell culture

The cell culture was performed using a protocol described previously in our studies^12,13^. A Caco-2 cell line (HTB-37), derived from a human colorectal adenocarcinoma (ATCC, Manassas, VA, USA), were grown (at passage number 29–45) in DMEM supplemented with 1% penicillin/streptomycin, 1% MEM non-essential amino acid and 10% FBS, further indicated as DMEM^+^.

The cells, in the microfluidic chip, were seeded at a density of 75,000 cell per cm^2^ in the devices and were allowed to attach to the membrane for 24 h, without the fluid flow. The membrane was then inserted in the microfluidic chip and cells were exposed to a continuous flow of 100 µL/h DMEM^+^ until day 21 of culturing (Fig. 1C). By doing so, the shear stress in the AP compartment was ~ 0.002 dyne/cm^2^ at the cell membrane area where the cells were grown. In vivo shear stress in the gut is reported to range between ~ 0.002 and 12.0 dyne/cm^2^^10,58,59^. The DMEM^+^ medium contained sodium bicarbonate (10 mM) to optimize the pH buffering capacity.

In Transwell, the cells were seeded at the same density as in the microfluidic chip (~ 75,000 cells per cm^2^) on 12-well Transwell PET inserts with pore size of 0.4 µm and surface area of 1.12 cm^2^ (Corning Amsterdam, The Netherlands) and cultured in DMEM^+^ for 21 days. The medium was replaced every two to three days.

### Fluorescent imaging of epithelial cell morphology

Morphological assessment of the Caco-2 cell monolayers, grown in the gut-on-chip or Transwell for 21 days, was performed as described previously in our studies^12,13^. In short, the cells were fixed with 4% formaldehyde for 10 min and rinsed with PBS at room temperature. Cells were then permeabilized with 0.25% Triton X100 in PBS for 10 min and blocked with 1% acetylated bovine serum albumin in PBS for 30 min. Conjugated antibody ZO-1/TJP1-Alexa Fluor 594 (Invitrogen, Waltham, MA) at 10 µg/mL was used to stain tight junctions. The nuclei were stained with 5 µg/mL DAPI (Invitrogen, Waltham, MA) and 4 U/mL Phalloidin Alexa Fluor 488 (Life technologies, Carlsbad, CA) was used to stain actin filaments (i.e. cytoskeleton). All stainings were incubated for 30 min. The membrane was then placed between two cover slips separated by a spacer (0.12 mm depth × 20 mm diameter) and a drop of anti-fading mounting medium was applied on the cells. The same staining procedure was used for the cells cultured on Transwell membranes. The stained monolayers of cells were analyzed using a confocal microscope (LSM 510 UVMETA; Carl Zeiss, Germany). Samples were excited with 405, 488 and 543 nm lasers. Multi-tracked images were captured to avoid bleed through. The used pinholes were in the range of 148–152 µm at a magnification of 40x. The gain and offset for the different channels were kept constant during the entire experiment.

### RNA isolation

Caco-2 cells were grown in the gut-on-chip or Transwell for 21 days. The chips were opened, and cells were washed with PBS. After that, 100 µL of RLT lysis buffer were added to the cell culture membrane and incubated for 1–2 min, then the membrane was rinsed with another 100 µL RLT lysis buffer. Cell lysates were then collected and the total RNA extraction was performed using the Qiagen RNAeasy Micro kit according to the manufacturer’s instructions. The RNA amount was determined using a Nanodrop (ND-1000 Thermoscientific Wilmington, Delaware, USA).

To the cells cultured on Transwell membranes 350 µL of RLT lysis buffer were added, cell lysates were then collected and analyzed using the same procedure.

### Affymetrix microarray processing, and analysis

The isolated RNA (n = 4 per group) was subjected to genome‐wide expression profiling. In brief, total RNA was labelled using the Whole-Transcript Sense Target Assay (Affymetrix, Santa Clara, CA, USA) and hybridized on human Gene 2.1 ST arrays (Affymetrix). The quality control and data analysis pipeline has been described in detail previously^60^. Normalized expression estimates of probe sets were computed by the robust multiarray analysis (RMA) algorithm^61,62^ as implemented in the Bioconductor library affyPLM. Probe sets were redefined using current genome definitions available from the NCBI database, which resulted in the profiling of 29,635 unique genes (custom CDF version 23)^63^. Differentially expressed probe sets (genes) were identified by using linear models (library limma) and an intensity-based moderated t-statistic^64,65^. Probe sets that satisfied the criterion of a False Discovery Rate (FDR) < 0.01 were considered to be significantly regulated^66^. Microarray data have been submitted to the Gene Expression Omnibus (accession number: GSE156269).

### Biological interpretation of array data

Changes in gene expression were related to biologically meaningful changes using gene set enrichment analysis (GSEA). It is well accepted that GSEA has multiple advantages over analyses performed on the level of individual genes^67–69^. GSEA evaluates gene expression on the level of gene sets that are based on prior biological knowledge, GSEA is unbiased, because no gene selection step (fold change and/or p-value cutoff) is used; a GSEA score is computed based on all genes in the gene set, which boosts the S/N ratio and allows to detect affected biological processes that are due to only subtle changes in expression of individual genes. Gene sets were retrieved from the expert‐curated KEGG database^70,71^, but sets belonging to the categories ‘6—Human Disease’ and ‘7—Drug Development’ (BRITE Functional Hierarchy level 1) were excluded. Moreover, only gene sets comprising more than 15 and fewer than 500 genes were taken into account. For each comparison, genes were ranked on their t‐value that was calculated by the moderated t‐test. Statistical significance of GSEA results was determined using 10,000 permutations.

### Comparison of Caco-2 and human in vivo gastrointestinal tract transcriptome data

To compare the transcriptome profiles of Caco-2 cells grown under dynamic (gut-on-chip) or static conditions (Transwell) with healthy human intestinal tissues, transcriptome data from 5 locations taken along the gastrointestinal tract (duodenum, jejunum, ileum, and colon) in 4 healthy human volunteers was used^22^. Datasets were integrated applying a cumulative proportion transformation using YuGene^72^, and visualized by principal component analysis (PCA), essentially as described before^73^. In brief, raw data transcriptome (CEL) files from the gastrointestinal were obtained from the Gene Expression Omnibus (GEO)^74^ (accession number: GSE10867). Next, each dataset was separately background corrected, log2-transformed and summarized at the probe set level, which was followed by filtering out all genes that were not shared on the two array platforms. Samples were then combined by rescaling using the cumulative proportion transformation. The combined dataset included the gene expression measurements of 12,746 genes in 22 samples. Before PCA, expression data was centered by dataset. PCA was performed and visualized using the library PCAtools^75^. A list of 764 intestine-specific genes was obtained from The Human Proteome Atlas^24^, and used when indicated.

## Supplementary Information

Supplementary Information

## References

[1] Martignoni M, Groothuis GMM, de Kanter R (2006). Species differences between mouse, rat, dog, monkey and human CYP-mediated drug metabolism, inhibition and induction. Expert. Opin. Drug Met..

[2] Komura H, Iwaki M (2011). In vitro and in vivo small intestinal metabolism of CYP3A and UGT substrates in preclinical animals species and humans: Species differences. Drug Metab. Rev..

[3] Punt A, Bouwmeester H, Schiffelers MWA, Peijnenburg A (2018). Expert opinions on the acceptance of alternative methods in food safety evaluations: Formulating recommendations to increase acceptance of non-animal methods for kinetics. Regul. Toxicol. Pharmacol..

[4] Flecknell P (2002). Replacement, reduction and refinement. Altex.

[5] Guerra A, Campillo NE, Paez JA (2010). Neural computational prediction of oral drug absorption based on CODES 2D descriptors. Eur. J. Med. Chem..

[6] Kampfer AAM (2017). Development of an in vitro co-culture model to mimic the human intestine in healthy and diseased state. Toxicol. In Vitro.

[7] Creff J (2019). Fabrication of 3D scaffolds reproducing intestinal epithelium topography by high-resolution 3D stereolithography. Biomaterials.

[8] Bhise NS (2014). Organ-on-a-chip platforms for studying drug delivery systems. J. Control Rel..

[9] Kimura H, Sakai Y, Fujii T (2018). Organ/body-on-a-chip based on microfluidic technology for drug discovery. Drug Metab. Pharmacokinet..

[10] Kim HJ, Huh D, Hamilton G, Ingber DE (2012). Human gut-on-a-chip inhabited by microbial flora that experiences intestinal peristalsis-like motions and flow. Lab Chip.

[11] Kim HJ, Ingber DE (2013). Gut-on-a-Chip microenvironment induces human intestinal cells to undergo villus differentiation. Integr. Biol. (Camb).

[12] Kulthong K (2018). Implementation of a dynamic intestinal gut-on-a-chip barrier model for transport studies of lipophilic dioxin congeners. RSC Adv..

[13] Kulthong K (2020). Microfluidic chip for culturing intestinal epithelial cell layers: Characterization and comparison of drug transport between dynamic and static models. Toxicol In Vitro.

[14] Ortmann D, Vallier L (2017). Variability of human pluripotent stem cell lines. Curr. Opin. Genet. Dev..

[15] Kasendra M (2018). Development of a primary human small intestine-on-a-chip using biopsy-derived organoids. Sci. Rep..

[16] del Carmen, P. M., Jean-Pierre, G. & Caroline, L. B. Intestinal in vitro cell culture models and their potential to study the effect of food components on intestinal inflammation (vol 59, pg 1, 2019). *Crit Rev Food Sci.* **59**, 2166–2168. doi:10.1080/10408398.2018.1543037 (2019). 10.1080/10408398.2018.1506734 30277794

[17] Punt A, Peijnenburg A, Hoogenboom R, Bouwmeester H (2017). Non-animal approaches for toxicokinetics in risk evaluations of food chemicals. Altex.

[18] Li C, Liu T, Cui X, Uss AS, Cheng KC (2007). Development of in vitro pharmacokinetic screens using Caco-2, human hepatocyte, and Caco-2/human hepatocyte hybrid systems for the prediction of oral bioavailability in humans. J. Biomol. Screen.

[19] Kim HJ, Li H, Collins JJ, Ingber DE (2016). Contributions of microbiome and mechanical deformation to intestinal bacterial overgrowth and inflammation in a human gut-on-a-chip. Proc. Natl. Acad. Sci. USA.

[20] Delon LC (2019). A systematic investigation of the effect of the fluid shear stress on Caco-2cells towards the optimization of epithelial organ-on-chip models. Biomaterials.

[21] Hubatsch I, Ragnarsson EG, Artursson P (2007). Determination of drug permeability and prediction of drug absorption in Caco-2 monolayers. Nat. Protoc..

[22] Comelli EM (2009). Biomarkers of human gastrointestinal tract regions. Mamm. Genome.

[23] R: A Language and Environment for Statistical Computing (R Foundation for Statistical Computing, Vienna, Austria, 2020).

[24] Uhlen, M. *et al.* Proteomics. Tissue-based map of the human proteome. *Science* **347**, 1260419. doi:10.1126/science.1260419 (2015). 10.1126/science.1260419 25613900

[25] Meunier V, Bourrie M, Berger Y, Fabre G (1995). The human intestinal epithelial cell line Caco-2; pharmacological and pharmacokinetic applications. Cell Biol. Toxicol..

[26] Sun H, Chow EC, Liu S, Du Y, Pang KS (2008). The Caco-2 cell monolayer: Usefulness and limitations. Expert Opin. Drug Metab. Toxicol..

[27] Wang Z, Litterio MC, Muller M, Vauzour D, Oteiza PI (2020). (-)-Epicatechin and NADPH oxidase inhibitors prevent bile acid-induced Caco-2 monolayer permeabilization through ERK1/2 modulation. Redox. Biol..

[28] Gayer CP, Basson MD (2009). The effects of mechanical forces on intestinal physiology and pathology. Cell Signal.

[29] Chen BP (2001). DNA microh shear stressarray analysis of gene expression in endothelial cells in response to 24-. Physiol. Genomics.

[30] Ohura N (2003). Global analysis of shear stress-responsive genes in vascular endothelial cells. J. Atheroscler. Thromb..

[31] Kunnen SJ, Malas TB, Semeins CM, Bakker AD, Peters DJM (2018). Comprehensive transcriptome analysis of fluid shear stress altered gene expression in renal epithelial cells. J. Cell. Physiol..

[32] Sigel, A., Sigel, H., Sigel, R. K. O. & Royal Society of Chemistry (Great Britain). *Metallothioneins Related chelators*. (RSC Pub., 2009).

[33] Kumari MV, Hiramatsu M, Ebadi M (1998). Free radical scavenging actions of metallothionein isoforms I and II. Free Radic. Res..

[34] Conway DE (2010). Endothelial metallothionein expression and intracellular free zinc levels are regulated by shear stress. Am. J. Physiol. Cell Physiol..

[35] Radominska-Pandya A, Czernik PJ, Little JM, Battaglia E, Mackenzie PI (1999). Structural and functional studies of UDP-glucuronosyltransferases. Drug Metab. Rev..

[36] Miners JO, Mackenzie PI (1991). Drug glucuronidation in humans. Pharmacol. Therapeut..

[37] Yueh MF, Bonzo JA, Tukey RH (2005). The role of ah receptor in induction of human UDP-glucuronosyltransferase 1A1. Method Enzymol..

[38] Brauze D (2017). Induction of expression of aryl hydrocarbon receptor-dependent genes in human HepaRG cell line modified by shRNA and treated with beta-naphthoflavone. Mol. Cell. Biochem..

[39] Han Z (2008). Aryl hydrocarbon receptor mediates laminar fluid shear stress-induced CYP1A1 activation and cell cycle arrest in vascular endothelial cells. Cardiovasc. Res..

[40] Lano G (2020). Aryl hydrocarbon receptor activation and tissue factor induction by fluid shear stress and indoxyl sulfate in endothelial cells. Int. J. Mol. Sci..

[41] Conway DE (2009). Expression of CYP1A1 and CYP1B1 in human endothelial cells: Regulation by fluid shear stress. Cardiovasc. Res..

[42] Chen G, Shaw MH, Kim YG, Nunez G (2009). NOD-like receptors: role in innate immunity and inflammatory disease. Annu. Rev. Pathol..

[43] Loo YM, Gale M (2011). Immune signaling by RIG-I-like receptors. Immunity.

[44] Miravete M (2012). Renal tubular fluid shear stress facilitates monocyte activation toward inflammatory macrophages. Am. J. Physiol. Renal. Physiol..

[45] Harrison, D. A. The Jak/STAT pathway. *Cold Spring Harb Perspect Biol* **4**. doi:10.1101/cshperspect.a011205 (2012). 10.1101/cshperspect.a011205 PMC3282412 22383755

[46] Eric Ho, J. W. in *Handbook of Cell Signaling* (ed Edward A. Dennis Ralph A. Bradshaw) Ch. 71, 533–538 (Academic Press, 2010).

[47] Veronica Lifshitz, D. F. in *Handbook of Biologically Active Peptides* (ed Abba J. Kastin) Ch. **225**, 1647–1653 (Academic Press, 2013).

[48] Varfolomeev E, Vucic D (2018). Intracellular regulation of TNF activity in health and disease. Cytokine.

[49] Mitchell S, Vargas J, Hoffmann A (2016). Signaling via the NFkappaB system. Wiley Interdiscip. Rev. Syst. Biol. Med..

[50] Cheng M, Wu J, Li Y, Nie Y, Chen H (2008). Activation of MAPK participates in low shear stress-induced IL-8 gene expression in endothelial cells. Clin. Biomech. (Bristol, Avon).

[51] Walshe TE (2013). The role of shear-induced transforming growth factor-beta signaling in the endothelium. Arterioscler. Thromb. Vasc. Biol..

[52] Hay DC (2003). Activation of NF-kappaB nuclear transcription factor by flow in human endothelial cells. Biochim. Biophys. Acta.

[53] Doddaballapur A (2015). Laminar shear stress inhibits endothelial cell metabolism via KLF2-mediated repression of PFKFB3. Arterioscler. Thromb. Vasc. Biol..

[54] Punt, A. *et al.* New approach methodologies (NAMs) for human-relevant biokinetics predictions. Meeting the paradigm shift in toxicology towards an animal-free chemical risk assessment. *ALTEX* **37**, 607–622. doi:10.14573/altex.2003242 (2020). 10.14573/altex.2003242 32521035

[55] Steinway SN, Saleh J, Koo BK, Delacour D, Kim DH (2020). Human microphysiological models of intestinal tissue and gut microbiome. Front. Bioeng. Biotechnol..

[56] Ashammakhi N (2020). Gut-on-a-chip: Current progress and future opportunities. Biomaterials.

[57] Hewes SA (2020). In vitro models of the small intestine: Engineering challenges and engineering solutions. Tissue Eng. Part B Rev..

[58] Guo P, Weinstein AM, Weinbaum S (2000). A hydrodynamic mechanosensory hypothesis for brush border microvilli. Am. J. Physiol. Renal. Physiol..

[59] Hardacre AK, Lentle RG, Yap S-Y, Monro JA (2016). Does viscosity or structure govern the rate at which starch granules are digested?. Carbohyd. Polym..

[60] Lin K (2011). MADMAX: Management and analysis database for multiple ~omics experiments. J. Integr. Bioinform..

[61] Irizarry RA (2003). Exploration, normalization, and summaries of high density oligonucleotide array probe level data. Biostatistics.

[62] Bolstad BM, Irizarry RA, Astrand M, Speed TP (2003). A comparison of normalization methods for high density oligonucleotide array data based on variance and bias. Bioinformatics.

[63] Dai MH (2005). Evolving gene/transcript definitions significantly alter the interpretation of GeneChip data. Nucleic Acids Res.

[64] Ritchie ME (2015). limma powers differential expression analyses for RNA-sequencing and microarray studies. Nucleic Acids Res..

[65] Sartor MA (2006). Intensity-based hierarchical Bayes method improves testing for differentially expressed genes in microarray experiments. BMC Bioinformatics.

[66] Benjamini Y, Hochberg Y (1995). Controlling the false discovery rate: A practical and powerful approach to multiple testing. J. R Stat. Soc. B.

[67] Subramanian A (2005). Gene set enrichment analysis: A knowledge-based approach for interpreting genome-wide expression profiles. Proc. Natl. Acad. Sci. USA.

[68] Allison DB, Cui X, Page GP, Sabripour M (2006). Microarray data analysis: from disarray to consolidation and consensus. Nat. Rev. Genet..

[69] Abatangelo L (2009). Comparative study of gene set enrichment methods. BMC Bioinformatics.

[70] Kanehisa M, Furumichi M, Tanabe M, Sato Y, Morishima K (2017). KEGG: New perspectives on genomes, pathways, diseases and drugs. Nucleic Acids Res..

[71] Kanehisa M, Goto S (2000). KEGG: kyoto encyclopedia of genes and genomes. Nucleic Acids Res..

[72] Le Cao KA, Rohart F, McHugh L, Korn O, Wells CA (2014). YuGene: a simple approach to scale gene expression data derived from different platforms for integrated analyses. Genomics.

[73] Rohart F (2016). A molecular classification of human mesenchymal stromal cells. Peer J..

[74] Clough E, Barrett T (2016). The gene expression omnibus database. Methods Mol. Biol..

[75] Blighe, K., *PCAtools**: **everything Principal Components Analysis*, https://github.com/kevinblighe/PCAtools (2018).

