# Supplementary material for: Transcriptome comparisons of in vitro intestinal epithelia grown under static and microfluidic gut-on-chip conditions with in vivo human epithelia
Source: Sci Rep. 2021 Feb 5;11:3234. doi: 10.1038/s41598-021-82853-6 (PMC7864925; doi:10.1038/s41598-021-82853-6)
Supplement: Supplementary file 1 — Supplementary Information [file 41598_2021_82853_MOESM1_ESM.pdf]

Transcriptome comparisons of *in vitro* intestinal epithelia grown under static and microfluidic gut-on-chip conditions with *in vivo* human epithelia

Kornphimol Kulthong, Guido J.E.J. Hooiveld, Loes Duivenvoorde, Ignacio Miro Estruch, Victor Marin, Meike van der Zande and Hans Bouwmeester

Supplementary Table S1. Upregulated pathways in Caco-2 cells cultured under dynamic culture conditions versus static culture conditions

KEGG pathway name	KEGG Category	KEGG category subgroup	SIZE	ES	NES	NOM p-val	FDR q-val
Ribosome biogenesis in eukaryotes	Genetic information processing	Translation	76	0.77	2.52	0.0E+00	0.0E+00
Proteasome	Genetic information processing	Folding, sorting and degradation	43	0.79	2.37	0.0E+00	0.0E+00
Spliceosome	Genetic information processing	Transcription	128	0.67	2.36	0.0E+00	0.0E+00
RNA transport	Genetic information processing	Translation	146	0.64	2.32	0.0E+00	0.0E+00
Protein processing in endoplasmic reticulum	Genetic information processing	Folding, sorting and degradation	160	0.60	2.18	0.0E+00	0.0E+00
RNA polymerase	Genetic information processing	Transcription	29	0.75	2.05	0.0E+00	8.7E-05
Cytosolic DNA sensing pathway	Organismal systems	Immune system	58	0.64	2.01	0.0E+00	1.7E-04
Antigen processing and presentation	Organismal systems	Immune system	67	0.62	2.01	0.0E+00	1.6E-04
TNF signaling pathway	Environmental information processing	Signal transduction	110	0.58	2.00	0.0E+00	2.2E-04
NF Kappa-B signaling pathway	Environmental information processing	Signal transduction	97	0.58	1.96	0.0E+00	3.5E-04
NOD-like receptor signaling pathway	Organismal systems	Immune system	168	0.53	1.93	0.0E+00	5.6E-04
Cell cycle	Cellular processes	Cell growth and death	124	0.55	1.92	0.0E+00	6.5E-04
mRNA surveillance pathway	Genetic information processing	Translation	87	0.54	1.82	0.0E+00	3.4E-03
Osteoclast differentiation	Organismal systems	Development and regeneration	122	0.51	1.81	0.0E+00	3.6E-03
Necroptosis	Cellular processes	Cell growth and death	128	0.50	1.77	0.0E+00	6.2E-03
Protein export	Genetic information processing	Folding, sorting and degradation	22	0.68	1.76	3.3E-03	6.7E-03
Snare interactions in vesicular transport	Genetic information processing	Folding, sorting and degradation	33	0.62	1.74	3.8E-03	8.1E-03
Mismatch repair	Genetic information processing	Replication and repair	23	0.66	1.74	4.1E-03	8.3E-03
Apoptosis multiple species	Cellular processes	Cell growth and death	31	0.62	1.73	4.7E-03	8.6E-03
IL-17 signaling pathway	Organismal systems	Immune system	93	0.51	1.72	3.7E-04	9.5E-03
p53 signaling pathway	Cellular processes	Cell growth and death	72	0.52	1.70	1.1E-03	1.1E-02
Basal transcription factors	Genetic information processing	Transcription	42	0.57	1.68	4.9E-03	1.3E-02
Cytokine-cytokine receptor interaction	Environmental information processing	Signaling molecules and interaction	284	0.43	1.67	0.0E+00	1.5E-02
Apoptosis	Cellular processes	Cell growth and death	133	0.47	1.66	1.8E-04	1.6E-02
Th17 cell differentiation	Organismal systems	Immune system	105	0.48	1.65	5.6E-04	1.6E-02
DNA replication	Genetic information processing	Replication and repair	36	0.56	1.61	9.8E-03	2.4E-02
Rig-I-like receptor signaling pathway	Organismal systems	Immune system	67	0.50	1.60	3.8E-03	2.8E-02
MAPK signaling pathway	Environmental information processing	Signal transduction	292	0.41	1.60	0.0E+00	2.7E-02
Progesterone mediated oocyte maturation	Organismal systems	Endocrine system	93	0.47	1.59	4.7E-03	2.7E-02
Nucleotide excision repair	Genetic information processing	Replication and repair	43	0.54	1.59	1.1E-02	2.7E-02
C-type lectin receptor signaling pathway	Organismal systems	Immune system	104	0.46	1.58	1.7E-03	2.8E-02
Synaptic vesicle cycle	Organismal systems	Nervous system	78	0.47	1.55	5.8E-03	3.5E-02

Estrogen signaling pathway	Environmental information processing	Signaling molecules and interaction	136	0.43	1.54	1.8E-03	3.8E-02
Hippo signaling pathway multiple species	Environmental information processing	Signal transduction	28	0.57	1.54	2.0E-02	3.7E-02
Autophagy other	Cellular processes	Transport and catabolism	31	0.56	1.54	2.2E-02	3.7E-02
Vasopressin regulated water reabsorption	Organismal systems	Excretory system	44	0.51	1.53	2.2E-02	3.9E-02
Th1 and Th2 cell differentiation	Organismal systems	Immune system	90	0.45	1.51	7.8E-03	4.7E-02
Collecting duct acid secretion	Organismal systems	Excretory system	27	0.56	1.49	3.6E-02	5.1E-02
RNA degradation	Genetic information processing	Folding, sorting and degradation	77	0.46	1.49	1.4E-02	5.2E-02
Oocyte meiosis	Cellular processes	Cell growth and death	119	0.42	1.48	6.3E-03	5.2E-02
Ubiquitin mediated proteolysis	Genetic information processing	Folding, sorting and degradation	136	0.41	1.48	4.6E-03	5.2E-02
TGF-beta signaling pathway	Environmental information processing	Signal transduction	92	0.43	1.47	1.3E-02	5.9E-02
Oxidative phosphorylation	Metabolism	Energy metabolism	115	0.41	1.44	1.3E-02	6.9E-02
Adipocytokine signaling pathway	Organismal systems	Endocrine system	68	0.44	1.43	2.8E-02	7.4E-02
Endocytosis	Cellular processes	Transport and catabolism	242	0.37	1.42	3.4E-03	7.8E-02
Jak- STAT signaling pathway	Environmental information processing	Signal transduction	158	0.39	1.41	1.1E-02	8.3E-02
Cellular senescence	Cellular processes	Cell growth and death	158	0.38	1.39	1.4E-02	9.3E-02
Hippo signaling pathway	Environmental information processing	Signal transduction	150	0.38	1.39	1.3E-02	9.6E-02
Viral protein interaction with cytokine and cytokine receptor	Environmental information processing	Signaling molecules and interaction	96	0.40	1.38	3.0E-02	9.8E-02
Phagosome	Cellular processes	Transport and catabolism	50	0.45	1.37	6.3E-02	1.1E-01
Autophagy animal	Cellular processes	Transport and catabolism	143	0.38	1.36	2.4E-02	1.1E-01
Thermogenesis	Organismal systems	Environmental adaptation	211	0.35	1.33	2.1E-02	1.3E-01

Supplementary Table S2. Downregulated pathways in Caco-2 cells cultured under dynamic culture conditions versus static culture conditions

KEGG pathway name	KEGG Category	KEGG category subgroup	SIZE	ES	NES	NOM p-val	FDR q-val
Protein digestion and absorption	Organismal systems	Digestive system	83	-0.66	-2.23	0.0E+00	1.1E-04
Carbohydrate digestion and absorption	Organismal systems	Digestive system	39	-0.75	-2.19	0.0E+00	5.7E-05
Fat digestion and absorption	Organismal systems	Digestive system	41	-0.73	-2.17	0.0E+00	3.8E-05
Glycolysis gluconeogenesis	Metabolism	Carbohydrate metabolism	68	-0.63	-2.05	0.0E+00	2.7E-04
Vitamin digestion and bsorption	Organismal systems	Digestive system	23	-0.76	-2.00	0.0E+00	4.8E-04
Carbon metabolism	Metabolism	Global and overview maps	114	-0.57	-2.00	0.0E+00	4.3E-04
Drug metabolism cytochrome P450	Metabolism	Xenobiotics biodegradation and metabolism	62	-0.62	-2.00	0.0E+00	4.4E-04
Fatty acid degradation	Metabolism	Lipid metabolism	43	-0.65	-1.98	0.0E+00	6.7E-04
Cholesterol metabolism	Organismal systems	Digestive system	49	-0.64	-1.96	0.0E+00	7.9E-04
Steroid biosynthesis	Metabolism	Lipid metabolism	18	-0.79	-1.96	2.1E-04	7.2E-04
Butanoate metabolism	Metabolism	Carbohydrate metabolism	28	-0.71	-1.96	4.2E-04	6.9E-04
Propanoate metabolism	Metabolism	Carbohydrate metabolism	33	-0.69	-1.95	2.1E-04	7.2E-04
Starch and sucrose metabolism	Metabolism	Carbohydrate metabolism	32	-0.67	-1.91	4.2E-04	1.3E-03

Glycine serine and threonine metabolism	Metabolism	Amino acid metabolism	38	-0.65	-1.91	4.3E-04	1.4E-03
Peroxisome	Cellular processes	Transport and catabolism	82	-0.56	-1.90	0.0E+00	1.4E-03
Valine leucine and isoleucine degradation	Metabolism	Amino acid metabolism	47	-0.62	-1.88	4.2E-04	1.9E-03
Pentose phosphate pathway	Metabolism	Carbohydrate metabolism	30	-0.67	-1.87	6.4E-04	2.2E-03
Histidine metabolism	Metabolism	Amino acid metabolism	23	-0.71	-1.87	1.1E-03	2.1E-03
Complement and coagulation cascades	Organismal systems	Immune system	77	-0.56	-1.86	2.1E-04	2.0E-03
Biosynthesis of amino acids	Metabolism	Global and overview maps	71	-0.57	-1.86	0.0E+00	2.2E-03
Glyoxylate and dicarboxylate metabolism	Metabolism	Carbohydrate metabolism	29	-0.67	-1.85	1.5E-03	2.3E-03
Primary bile acid biosynthesis	Metabolism	Lipid metabolism	16	-0.76	-1.83	1.9E-03	3.2E-03
Bile secretion	Organismal systems	Digestive system	71	-0.54	-1.79	6.4E-04	5.2E-03
Tyrosine metabolism	Metabolism	Amino acid metabolism	36	-0.61	-1.77	1.5E-03	6.4E-03
Sphingolipid metabolism	Metabolism	Lipid metabolism	46	-0.58	-1.76	1.7E-03	6.9E-03
Galactose metabolism	Metabolism	Carbohydrate metabolism	31	-0.62	-1.73	4.9E-03	8.8E-03
PPAR signaling pathway	Organismal systems	Endocrine system	73	-0.52	-1.70	6.4E-04	1.2E-02
Fructose and mannose metabolism	Metabolism	Carbohydrate metabolism	32	-0.60	-1.69	4.6E-03	1.3E-02
Proximal tubule bicarbonate reclamation	Organismal systems	Excretory system	22	-0.64	-1.66	8.9E-03	1.7E-02
Nitrogen metabolism	Metabolism	Energy metabolism	16	-0.69	-1.66	1.1E-02	1.8E-02
Inositol phosphate metabolism	Metabolism	Carbohydrate metabolism	74	-0.50	-1.65	1.1E-03	2.0E-02
Tryptophan metabolism	Metabolism	Amino acid metabolism	42	-0.55	-1.64	7.5E-03	2.0E-02
Alanine, aspartate and glutamate metabolism	Metabolism	Amino acid metabolism	34	-0.57	-1.62	1.3E-02	2.4E-02
Fatty acid metabolism	Metabolism	Global and overview maps	55	-0.52	-1.61	5.3E-03	2.5E-02
Phosphatidylinositol signaling system	Environmental information processing	Signal transduction	99	-0.46	-1.60	2.2E-03	2.9E-02
Pancreatic secretion	Organismal systems	Digestive system	93	-0.46	-1.57	3.0E-03	3.7E-02
Citrate cycle (TCA cycle)	Metabolism	Carbohydrate metabolism	29	-0.56	-1.56	2.3E-02	3.8E-02
Glucagon signaling pathway	Organismal systems	Endocrine system	100	-0.45	-1.56	3.9E-03	3.7E-02
Glycerolipid metabolism	Metabolism	Lipid metabolism	61	-0.49	-1.55	8.2E-03	3.8E-02
Glycerophospholipid metabolism	Metabolism	Lipid metabolism	93	-0.45	-1.55	4.0E-03	4.0E-02
Pyruvate metabolism	Metabolism	Carbohydrate metabolism	39	-0.53	-1.54	2.5E-02	3.9E-02
Glycosaminoglycan biosynthesis-heparan sulfate/heparin	Metabolism	Glycan biosynthesis and metabolism	24	-0.58	-1.54	2.7E-02	4.1E-02
Phenylalanine metabolism	Metabolism	Amino acid metabolism	17	-0.62	-1.53	4.0E-02	4.3E-02
Oxocarboxylic acid metabolism	Metabolism	Global and overview maps	16	-0.62	-1.51	4.8E-02	4.8E-02
Linoleic acid metabolism	Metabolism	Lipid metabolism	25	-0.56	-1.50	3.9E-02	5.0E-02
Alpha linolenic acid metabolism	Metabolism	Lipid metabolism	22	-0.57	-1.50	4.4E-02	5.2E-02
ABC transporters	Environmental information processing	Membrane transport	45	-0.49	-1.48	2.9E-02	5.8E-02
Lysosome	Cellular processes	Transport and catabolism	122	-0.41	-1.48	9.3E-03	5.9E-02
Arachidonic acid metabolism	Metabolism	Lipid metabolism	58	-0.47	-1.47	2.3E-02	6.1E-02
Drug metabolism other enzymes	Metabolism	Xenobiotics biodegradation and metabolism	70	-0.43	-1.42	2.5E-02	8.5E-02
Ether lipid metabolism	Metabolism	Lipid metabolism	44	-0.47	-1.42	4.5E-02	8.5E-02
AMPK signaling pathway	Environmental information processing	Signal transduction	119	-0.39	-1.39	2.1E-02	9.9E-02
Serotonergic synapse	Organismal systems	Nervous system	109	-0.39	-1.38	2.8E-02	1.1E-01

Insulin secretion	Organismal systems	Endocrine system	85	-0.40	-1.35	4.6E-02	1.3E-01
cGMP-PKG signaling pathway	Environmental information processing	Signal transduction	164	-0.35	-1.29	3.8E-02	1.7E-01
Neuroactive ligand-receptor interaction	Environmental information processing	Signaling molecules and interaction	330	-0.32	-1.28	1.7E-02	1.8E-01

Supplementary Table S3. References describing the function of genes in the top 10 most significantly up and downregulated genes

Genes	Core function in cells	NCBI Database[1]	KEGG Pathway Database[2]	Publications
MT1H	Mineral absorption, Metal binding	X	X	[1-3]
MT1G	Mineral absorption, Metal binding	X	X	[1-3]
ANXA1	Anti-inflammation	X	X	[4, 5]
UGT1A1	Metabolism	X	X	[6-8]
DHRS2	Metabolism	X	X	[9, 10]
MT1X	Mineral absorption, Metal binding	X	X	[1-3]
IFITM3	Immune system	X	X	[11, 12]
SLC16A6	Membrane transporter	X	X	[13, 14]
UPK1B	Cellular development and growth	X	X	[15]
IFI27	Immune system	X	NA	[16-18]
G6PC	Metabolism	X	X	[19, 20]
ALDOB	Metabolism	X	X	[21-23]
C17orf78	unknown	NA	NA	-
ASAH2	Metabolism, Biosynthesis	X	X	[24, 25]
SI	Metabolism, Digestion	X	X	[26-28]
S100G	Mineral absorption, Membrane transporter	X	X	[29-31]
CYP2C19	Metabolism	X	X	[32, 33]
MTTP	Lipid digestion and absorption	X	X	[34-36]
TM4SF20	Cell growth, proliferation and activities	X	NA	[37, 38]
NRN1	Neurite outgrowth	X	NA	[39, 40]

[1]https://www.ncbi.nlm.nih.gov/gene

[2]https://www.genome.jp/kegg/genes.html

1. Dziegiel, P., et al., *Metallothioneins in Normal and Cancer Cells.* Adv Anat Embryol Cell Biol, 2016. **218**: p. 1-117.
2. Subramanian Vignesh, K. and G.S. Deepe, Jr., *Metallothioneins: Emerging Modulators in Immunity and Infection.* Int J Mol Sci, 2017. **18**(10).
3. Cheng, Z.Q., et al., *Evaluation of metallothionein formation as a proxy for zinc absorption in an in vitro digestion/Caco-2 cell culture model.* Food & Function, 2012. **3**(7): p. 732-736.
4. Babbin, B.A., et al., *Annexin A1 regulates intestinal mucosal injury, inflammation, and repair.* J Immunol, 2008. **181**(7): p. 5035-44.
5. Gavins, F.N.E. and M.J. Hickey, *Annexin A1 and the regulation of innate and adaptive immunity.* Frontiers in Immunology, 2012. **3**.
6. Mackenzie, P.I., D.G. Hu, and D.A. Gardner-Stephen, *The regulation of UDP-glucuronosyltransferase genes by tissue-specific and ligand-activated transcription factors.* Drug Metab Rev, 2010. **42**(1): p. 99-109.
7. Vaessen, S.F., et al., *Regional Expression Levels of Drug Transporters and Metabolizing Enzymes along the Pig and Human Intestinal Tract and Comparison with Caco-2 Cells.* Drug Metab Dispos, 2017. **45**(4): p. 353-360.
8. Paine, M.F. and M.B. Fisher, *Immunochemical identification of UGT isoforms in human small bowel and in caco-2 cell monolayers.* Biochem Biophys Res Commun, 2000. **273**(3): p. 1053-7.
9. Gabrielli, F. and S. Tofanelli, *Molecular and functional evolution of human DHRS2 and DHRS4 duplicated genes.* Gene, 2012. **511**(2): p. 461-9.
10. Luo, X., et al., *DHRS2 mediates cell growth inhibition induced by Trichothecin in nasopharyngeal carcinoma.* J Exp Clin Cancer Res, 2019. **38**(1): p. 300.
11. Gorman, M.J., et al., *The Interferon-Stimulated Gene Ifitm3 Restricts West Nile Virus Infection and Pathogenesis.* J Virol, 2016. **90**(18): p. 8212-25.
12. Feeley, E.M., et al., *IFITM3 inhibits influenza A virus infection by preventing cytosolic entry.* PLoS Pathog, 2011. **7**(10): p. e1002337.
13. Halestrap, A.P. and D. Meredith, *The SLC16 gene family-from monocarboxylate transporters (MCTs) to aromatic amino acid transporters and beyond.* Pflugers Arch, 2004. **447**(5): p. 619-28.
14. Perez-Escuredo, J., et al., *Monocarboxylate transporters in the brain and in cancer.* Biochim Biophys Acta, 2016. **1863**(10): p. 2481-97.
15. Carpenter, A.R., et al., *Uroplakin 1b is critical in urinary tract development and urothelial differentiation and homeostasis.* Kidney Int, 2016. **89**(3): p. 612-24.
16. Dooley, T.P., et al., *Regulation of gene expression in inflammatory bowel disease and correlation with IBD drugs: screening by DNA microarrays.* Inflamm Bowel Dis, 2004. **10**(1): p. 1-14.
17. Nagasawa, Y., et al., *IFI27 Is a Useful Genetic Marker for Diagnosis of Immunoglobulin A Nephropathy and Membranous Nephropathy Using Peripheral Blood.* Plos One, 2016. **11**(4).
18. Kraiczy, J., et al., *Genome-Wide Epigenetic and Transcriptomic Characterization of Human-Induced Pluripotent Stem Cell-Derived Intestinal Epithelial Organoids.* Cell Mol Gastroenterol Hepatol, 2019. **7**(2): p. 285-288.
19. Chou, J.Y. and B.C. Mansfield, *Mutations in the glucose-6-phosphatase-alpha (G6PC) gene that cause type Ia glycogen storage disease.* Human Mutation, 2008. **29**(7): p. 921-930.
20. Guo, T., et al., *Genetic and molecular analyses reveal G6PC as a key element connecting glucose metabolism and cell cycle control in ovarian cancer.* Tumor Biology, 2015. **36**(10): p. 7649-7658.
21. Chang, J., et al., *Proteomic changes during intestinal cell maturation in vivo.* J Proteomics, 2008. **71**(5): p. 530-46.
22. Chang, Y.C., et al., *Roles of Aldolase Family Genes in Human Cancers and Diseases.* Trends Endocrinol Metab, 2018. **29**(8): p. 549-559.
23. Eady, J.J., et al., *Differential effects of basolateral and apical iron supply on iron transport in Caco-2 cells.* Genes Nutr, 2015. **10**(3): p. 463.
24. Kono, M., et al., *Neutral ceramidase encoded by the Asah2 gene is essential for the intestinal degradation of sphingolipids.* J Biol Chem, 2006. **281**(11): p. 7324-31.
25. Zhu, D., et al., *Expression and regulation of enzymes in the ceramide metabolic pathway in human retinal pigment epithelial cells and their relevance to retinal degeneration.* Vision Res, 2010. **50**(7): p. 643-51.
26. Hoffman, L.R. and E.B. Chang, *Regional Expression and Regulation of Intestinal Sucrase-Isomaltase.* Journal of Nutritional Biochemistry, 1993. **4**(3): p. 130-142.
27. Inoue, S., K. Mochizuki, and T. Goda, *Jejunal induction of SI and SGLT1 genes in rats by high-starch/low-fat diet is associated with histone acetylation and binding of GCN5 on the genes.* J Nutr Sci Vitaminol (Tokyo), 2011. **57**(2): p. 162-9.
28. Gericke, B., M. Amiri, and H.Y. Naim, *The multiple roles of sucrase-isomaltase in the intestinal physiology.* Mol Cell Pediatr, 2016. **3**(1): p. 2.
29. Hulst, M., et al., *Enrichment of in vivo transcription data from dietary intervention studies with in vitro data provides improved insight into gene regulation mechanisms in the intestinal mucosa.* Genes Nutr, 2017. **12**: p. 11.
30. Choi, K.C. and E.B. Jeung, *Molecular mechanism of regulation of the calcium-binding protein calbindin-D9k, and its physiological role(s) in mammals: a review of current research.* J Cell Mol Med, 2008. **12**(2): p. 409-20.
31. Ishiguro, K., et al., *S100G expression and function in fibroblasts on colitis induction.* Int Immunopharmacol, 2016. **39**: p. 92-96.
32. Galetin, A. and J.B. Houston, *Intestinal and hepatic metabolic activity of five cytochrome P450 enzymes: impact on prediction of first-pass metabolism.* J Pharmacol Exp Ther, 2006. **318**(3): p. 1220-9.
33. Hosohata, K., et al., *Impact of intestinal CYP2C19 genotypes on the interaction between tacrolimus and omeprazole, but not lansoprazole, in adult living-donor liver transplant patients.* Drug Metab Dispos, 2009. **37**(4): p. 821-6.
34. Xie, Y., et al., *Compensatory increase in hepatic lipogenesis in mice with conditional intestine-specific Mttp deficiency.* J Biol Chem, 2006. **281**(7): p. 4075-86.
35. Hussain, M.M., N. Nijstad, and L. Franceschini, *Regulation of microsomal triglyceride transfer protein.* Clin Lipidol, 2011. **6**(3): p. 293-303.
36. Liu, Y., et al., *Lack of MTTP Activity in Pluripotent Stem Cell-Derived Hepatocytes and Cardiomyocytes Abolishes apoB Secretion and Increases Cell Stress.* Cell Rep, 2017. **19**(7): p. 1456-1466.
37. Chen, Q., et al., *Sustained induction of collagen synthesis by TGF-beta requires regulated intramembrane proteolysis of CREB3L1.* PLoS One, 2014. **9**(10): p. e108528.
38. Denard, B., et al., *Ceramide Induced Regulated Alternative Translocation of TM4SF20 and Beyond.* Faseb Journal, 2017. **31**.
39. Gao, R., et al., *MiR-204 promotes apoptosis in oxidative stress-induced rat Schwann cells by suppressing neuritin expression.* FEBS Lett, 2014. **588**(17): p. 3225-32.
40. Feng, Y.A., T.E. Liu, and Y. Wu, *microRNA-182 inhibits the proliferation and migration of glioma cells through the induction of neuritin expression.* Oncol Lett, 2015. **10**(2): p. 1197-1203.

Supplementary Table S4. The names of the 575 genes that were visualized in Figure 4

ENTREZID	SYMBOL	GENENAME
3315	HSPB1	heat shock protein family B (small) member 1
2316	FLNA	filamin A
6237	RRAS	RAS related
4804	NGFR	nerve growth factor receptor
2318	FLNC	filamin C
4616	GADD45B	growth arrest and DNA damage inducible beta
374	AREG	amphiregulin
1326	MAP3K8	mitogen-activated protein kinase kinase kinase 8
4254	KITLG	KIT ligand
4791	NFKB2	nuclear factor kappa B subunit 2
1847	DUSP5	dual specificity phosphatase 5
1843	DUSP1	dual specificity phosphatase 1
3303	HSPA1A	heat shock protein family A (Hsp70) member 1A
3164	NR4A1	nuclear receptor subfamily 4 group A member 1
3725	JUN	Jun proto-oncogene, AP-1 transcription factor subunit
1849	DUSP7	dual specificity phosphatase 7
1649	DDIT3	DNA damage inducible transcript 3
3312	HSPA8	heat shock protein family A (Hsp70) member 8
9020	MAP3K14	mitogen-activated protein kinase kinase kinase 14
1969	EPHA2	EPH receptor A2
5536	PPP5C	protein phosphatase 5 catalytic subunit
3304	HSPA1B	heat shock protein family A (Hsp70) member 1B
9064	MAP3K6	mitogen-activated protein kinase kinase kinase 6
5922	RASA2	RAS p21 protein activator 2
9448	MAP4K4	mitogen-activated protein kinase kinase kinase kinase 4
11221	DUSP10	dual specificity phosphatase 10
2317	FLNB	filamin B
5604	MAP2K1	mitogen-activated protein kinase kinase 1
1435	CSF1	colony stimulating factor 1
5970	RELA	RELA proto-oncogene, NF-kB subunit
7040	TGFB1	transforming growth factor beta 1
1846	DUSP4	dual specificity phosphatase 4
5871	MAP4K2	mitogen-activated protein kinase kinase kinase kinase 2
1436	CSF1R	colony stimulating factor 1 receptor
2069	EREG	epiregulin
8605	PLA2G4C	phospholipase A2 group IVC
2260	FGFR1	fibroblast growth factor receptor 1
7124	TNF	tumor necrosis factor
7042	TGFB2	transforming growth factor beta 2
8817	FGF18	fibroblast growth factor 18
5971	RELB	RELB proto-oncogene, NF-kB subunit
5062	PAK2	p21 (RAC1) activated kinase 2
994	CDC25B	cell division cycle 25B
27092	CACNG4	calcium voltage-gated channel auxiliary subunit gamma 4
1848	DUSP6	dual specificity phosphatase 6
1844	DUSP2	dual specificity phosphatase 2
6788	STK3	serine/threonine kinase 3
4214	MAP3K1	mitogen-activated protein kinase kinase kinase 1
3480	IGF1R	insulin like growth factor 1 receptor

4215	MAP3K3	mitogen-activated protein kinase kinase kinase 3
7048	TGFBR2	transforming growth factor beta receptor 2
5155	PDGFB	platelet derived growth factor subunit B
4208	MEF2C	myocyte enhancer factor 2C
6722	SRF	serum response factor
1147	CHUK	conserved helix-loop-helix ubiquitous kinase
255189	PLA2G4F	phospholipase A2 group IVF
2321	FLT1	fms related tyrosine kinase 1
1647	GADD45A	growth arrest and DNA damage inducible alpha
25780	RASGRP3	RAS guanyl releasing protein 3
4790	NFKB1	nuclear factor kappa B subunit 1
7186	TRAF2	TNF receptor associated factor 2
8986	RPS6KA4	ribosomal protein S6 kinase A4
207	AKT1	AKT serine/threonine kinase 1
57551	TAOK1	TAO kinase 1
5534	PPP3R1	protein phosphatase 3 regulatory subunit B, alpha
9175	MAP3K13	mitogen-activated protein kinase kinase kinase 13
3554	IL1R1	interleukin 1 receptor type 1
51701	NLK	nemo like kinase
6300	MAPK12	mitogen-activated protein kinase 12
1616	DAXX	death domain associated protein
1845	DUSP3	dual specificity phosphatase 3
10912	GADD45G	growth arrest and DNA damage inducible gamma
836	CASP3	caspase 3
5923	RASGRF1	Ras protein specific guanine nucleotide releasing factor 1
51135	IRAK4	interleukin 1 receptor associated kinase 4
5908	RAP1B	RAP1B, member of RAS oncogene family
1956	EGFR	epidermal growth factor receptor
5567	PRKACB	protein kinase cAMP-activated catalytic subunit beta
5578	PRKCA	protein kinase C alpha
3625	INHBB	inhibin subunit beta B
26585	GREM1	gremlin 1, DAN family BMP antagonist
654	BMP6	bone morphogenetic protein 6
90	ACVR1	activin A receptor type 1
655	BMP7	bone morphogenetic protein 7
57154	SMURF1	SMAD specific E3 ubiquitin protein ligase 1
7057	THBS1	thrombospondin 1
3399	ID3	inhibitor of DNA binding 3, HLH protein
3397	ID1	inhibitor of DNA binding 1, HLH protein
1874	E2F4	E2F transcription factor 4
7050	TGIF1	TGFB induced factor homeobox 1
25805	BAMBI	BMP and activin membrane bound inhibitor
5516	PPP2CB	protein phosphatase 2 catalytic subunit beta
64750	SMURF2	SMAD specific E3 ubiquitin protein ligase 2
2033	EP300	E1A binding protein p300
4052	LTBP1	latent transforming growth factor beta binding protein 1
5515	PPP2CA	protein phosphatase 2 catalytic subunit alpha
6198	RPS6KB1	ribosomal protein S6 kinase B1
4089	SMAD4	SMAD family member 4
4170	MCL1	MCL1, BCL2 family apoptosis regulator
3459	IFNGR1	interferon gamma receptor 1

3455	IFNAR2	interferon alpha and beta receptor subunit 2
2475	MTOR	mechanistic target of rapamycin kinase
3953	LEPR	leptin receptor
3566	IL4R	interleukin 4 receptor
3572	IL6ST	interleukin 6 signal transducer
9021	SOCS3	suppressor of cytokine signaling 3
6774	STAT3	signal transducer and activator of transcription 3
53833	IL20RB	interleukin 20 receptor subunit beta
10254	STAM2	signal transducing adaptor molecule 2
3977	LIFR	LIF receptor alpha
51561	IL23A	interleukin 23 subunit alpha
3717	JAK2	Janus kinase 2
3976	LIF	LIF, interleukin 6 family cytokine
3563	IL3RA	interleukin 3 receptor subunit alpha
10379	IRF9	interferon regulatory factor 9
6776	STAT5A	signal transducer and activator of transcription 5A
8651	SOCS1	suppressor of cytokine signaling 1
6772	STAT1	signal transducer and activator of transcription 1
3561	IL2RG	interleukin 2 receptor subunit gamma
3601	IL15RA	interleukin 15 receptor subunit alpha
598	BCL2L1	BCL2 like 1
6773	STAT2	signal transducer and activator of transcription 2
1438	CSF2RA	colony stimulating factor 2 receptor alpha subunit
3589	IL11	interleukin 11
3567	IL5	interleukin 5
1271	CNTFR	ciliary neurotrophic factor receptor
8835	SOCS2	suppressor of cytokine signaling 2
3716	JAK1	Janus kinase 1
282618	IFNL1	interferon lambda 1
1154	CISH	cytokine inducible SH2 containing protein
5771	PTPN2	protein tyrosine phosphatase, non-receptor type 2
5290	PIK3CA	phosphatidylinositol-4,5-bisphosphate 3-kinase catalytic subunit alpha
595	CCND1	cyclin D1
2885	GRB2	growth factor receptor bound protein 2
4609	MYC	MYC proto-oncogene, bHLH transcription factor
3595	IL12RB2	interleukin 12 receptor subunit beta 2
282617	IFNL3	interferon lambda 3
896	CCND3	cyclin D3
5894	RAF1	Raf-1 proto-oncogene, serine/threonine kinase
3588	IL10RB	interleukin 10 receptor subunit beta
2689	GH2	growth hormone 2
8027	STAM	signal transducing adaptor molecule
7297	TYK2	tyrosine kinase 2
9306	SOCS6	suppressor of cytokine signaling 6
3592	IL12A	interleukin 12A
3383	ICAM1	intercellular adhesion molecule 1
330	BIRC3	baculoviral IAP repeat containing 3
5743	PTGS2	prostaglandin-endoperoxide synthase 2
1540	CYLD	CYLD lysine 63 deubiquitinase
5328	PLAU	plasminogen activator, urokinase
4792	NFKBIA	NFKB inhibitor alpha

4050	LTB	lymphotoxin beta
7706	TRIM25	tripartite motif containing 25
5588	PRKCQ	protein kinase C theta
7128	TNFAIP3	TNF alpha induced protein 3
1896	EDA	ectodysplasin A
7188	TRAF5	TNF receptor associated factor 5
7329	UBE2I	ubiquitin conjugating enzyme E2 I
8915	BCL10	B cell CLL/lymphoma 10
4067	LYN	LYN proto-oncogene, Src family tyrosine kinase
142	PARP1	poly(ADP-ribose) polymerase 1
3929	LBP	lipopolysaccharide binding protein
1906	EDN1	endothelin 1
3726	JUNB	JunB proto-oncogene, AP-1 transcription factor subunit
182	JAG1	jagged 1
7133	TNFRSF1B	TNF receptor superfamily member 1B
10488	CREB3	cAMP responsive element binding protein 3
3659	IRF1	interferon regulatory factor 1
192111	PGAM5	PGAM family member 5, mitochondrial serine/threonine protein phosphatase
3627	CXCL10	C-X-C motif chemokine ligand 10
6352	CCL5	C-C motif chemokine ligand 5
6364	CCL20	C-C motif chemokine ligand 20
2919	CXCL1	C-X-C motif chemokine ligand 1
10059	DNM1L	dynamin 1 like
90993	CREB3L1	cAMP responsive element binding protein 3 like 1
197259	MLKL	mixed lineage kinase domain like pseudokinase
4318	MMP9	matrix metallopeptidase 9
9586	CREB5	cAMP responsive element binding protein 5
1051	CEBPB	CCAAT enhancer binding protein beta
843	CASP10	caspase 10
8737	RIPK1	receptor interacting serine/threonine kinase 1
7533	YWHAH	tyrosine 3-monooxygenase/tryptophan 5-monooxygenase activation protein [illegible]
898	CCNE1	cyclin E1
9134	CCNE2	cyclin E2
8697	CDC23	cell division cycle 23
2810	SFN	stratifin
9184	BUB3	BUB3, mitotic checkpoint protein
25847	ANAPC13	anaphase promoting complex subunit 13
7532	YWHAG	tyrosine 3-monooxygenase/tryptophan 5-monooxygenase activation protein [illegible]
2932	GSK3B	glycogen synthase kinase 3 beta
51434	ANAPC7	anaphase promoting complex subunit 7
5111	PCNA	proliferating cell nuclear antigen
7465	WEE1	WEE1 G2 checkpoint kinase
5001	ORC5	origin recognition complex subunit 5
23595	ORC3	origin recognition complex subunit 3
991	CDC20	cell division cycle 20
990	CDC6	cell division cycle 6
8243	SMC1A	structural maintenance of chromosomes 1A
9088	PKMYT1	protein kinase, membrane associated tyrosine/threonine 1
4085	MAD2L1	mitotic arrest deficient 2 like 1
4173	MCM4	minichromosome maintenance complex component 4
993	CDC25A	cell division cycle 25A

545	ATR	ATR serine/threonine kinase
10393	ANAPC10	anaphase promoting complex subunit 10
1022	CDK7	cyclin dependent kinase 7
891	CCNB1	cyclin B1
699	BUB1	BUB1 mitotic checkpoint serine/threonine kinase
10926	DBF4	DBF4 zinc finger
7529	YWHAB	tyrosine 3-monooxygenase/tryptophan 5-monooxygenase activation protein
996	CDC27	cell division cycle 27
1871	E2F3	E2F transcription factor 3
5591	PRKDC	protein kinase, DNA-activated, catalytic subunit
1021	CDK6	cyclin dependent kinase 6
983	CDK1	cyclin dependent kinase 1
1028	CDKN1C	cyclin dependent kinase inhibitor 1C
7272	TTK	TTK protein kinase
9133	CCNB2	cyclin B2
246184	CDC26	cell division cycle 26
5347	PLK1	polo like kinase 1
1032	CDKN2D	cyclin dependent kinase inhibitor 2D
23594	ORC6	origin recognition complex subunit 6
1869	E2F1	E2F transcription factor 1
29945	ANAPC4	anaphase promoting complex subunit 4
1030	CDKN2B	cyclin dependent kinase inhibitor 2B
10274	STAG1	stromal antigen 1
10459	MAD2L2	mitotic arrest deficient 2 like 2
4176	MCM7	minichromosome maintenance complex component 7
8318	CDC45	cell division cycle 45
6500	SKP1	S-phase kinase associated protein 1
10744	PTTG2	pituitary tumor-transforming 2
701	BUB1B	BUB1 mitotic checkpoint serine/threonine kinase B
1019	CDK4	cyclin dependent kinase 4
5366	PMAIP1	phorbol-12-myristate-13-acetate-induced protein 1
1965	EIF2S1	eukaryotic translation initiation factor 2 subunit alpha
2081	ERN1	endoplasmic reticulum to nucleus signaling 1
9451	EIF2AK3	eukaryotic translation initiation factor 2 alpha kinase 3
8795	TNFRSF10B	TNF receptor superfamily member 10b
1520	CTSS	cathepsin S
6709	SPTAN1	spectrin alpha, non-erythrocytic 1
1514	CTSL	cathepsin L
84823	LMNB2	lamin B2
56616	DIABLO	diablo IAP-binding mitochondrial protein
10038	PARP2	poly(ADP-ribose) polymerase 2
637	BID	BH3 interacting domain death agonist
7277	TUBA4A	tubulin alpha 4a
4000	LMNA	lamin A/C
824	CAPN2	calpain 2
842	CASP9	caspase 9
3708	ITPR1	inositol 1,4,5-trisphosphate receptor type 1
51807	TUBA8	tubulin alpha 8
1515	CTSV	cathepsin V
27113	BBC3	BCL2 binding component 3
5599	MAPK8	mitogen-activated protein kinase 8

332	BIRC5	baculoviral IAP repeat containing 5
7846	TUBA1A	tubulin alpha 1a
1676	DFFA	DNA fragmentation factor subunit alpha
113457	TUBA3D	tubulin alpha 3d
71	ACTG1	actin gamma 1
839	CASP6	caspase 6
1512	CTSH	cathepsin H
1508	CTSB	cathepsin B
8878	SQSTM1	sequestosome 1
2512	FTL	ferritin light chain
5834	PYGB	glycogen phosphorylase B
834	CASP1	caspase 1
5481	PPID	peptidylprolyl isomerase D
3326	HSP90AB1	heat shock protein 90 alpha family class B member 1
9825	SPATA2	spermatogenesis associated 2
11124	FAF1	Fas associated factor 1
3320	HSP90AA1	heat shock protein 90 alpha family class A member 1
51510	CHMP5	charged multivesicular body protein 5
291	SLC25A4	solute carrier family 25 member 4
27183	VPS4A	vacuolar protein sorting 4 homolog A
5610	EIF2AK2	eukaryotic translation initiation factor 2 alpha kinase 2
91782	CHMP7	charged multivesicular body protein 7
29082	CHMP4A	charged multivesicular body protein 4A
7419	VDAC3	voltage dependent anion channel 3
55072	RNF31	ring finger protein 31
3552	IL1A	interleukin 1 alpha
81030	ZBP1	Z-DNA binding protein 1
5054	SERPINE1	serpin family E member 1
55240	STEAP3	STEAP3 metalloreductase
9538	EI24	EI24, autophagy associated transmembrane protein
3486	IGFBP3	insulin like growth factor binding protein 3
3732	CD82	CD82 molecule
51246	SHISA5	shisa family member 5
6241	RRM2	ribonucleotide reductase regulatory subunit M2
50484	RRM2B	ribonucleotide reductase regulatory TP53 inducible subunit M2B
92344	GORAB	golgin, RAB6 interacting
901	CCNG2	cyclin G2
2113	ETS1	ETS proto-oncogene 1, transcription factor
90550	MCU	mitochondrial calcium uniporter
677	ZFP36L1	ZFP36 ring finger protein like 1
4683	NBN	nibrin
91750	LIN52	lin-52 DREAM MuvB core complex component
5499	PPP1CA	protein phosphatase 1 catalytic subunit alpha
3133	HLA-E	major histocompatibility complex, class I, E
144715	RAD9B	RAD9 checkpoint clamp component B
55957	LIN37	lin-37 DREAM MuvB core complex component
4361	MRE11	MRE11 homolog, double strand break repair nuclease
23411	SIRT1	sirtuin 1
3106	HLA-B	major histocompatibility complex, class I, B
286826	LIN9	lin-9 DREAM MuvB core complex component
3105	HLA-A	major histocompatibility complex, class I, A

9110	MTMR4	myotubularin related protein 4
1606	DGKA	diacylglycerol kinase alpha
253430	IPMK	inositol polyphosphate multikinase
23236	PLCB1	phospholipase C beta 1
5295	PIK3R1	phosphoinositide-3-kinase regulatory subunit 1
54928	IMPAD1	inositol monophosphatase domain containing 1
5331	PLCB3	phospholipase C beta 3
8760	CDS2	CDP-diacylglycerol synthase 2
1040	CDS1	CDP-diacylglycerol synthase 1
3613	IMPA2	inositol monophosphatase 2
27124	INPP5J	inositol polyphosphate-5-phosphatase J
8821	INPP4B	inositol polyphosphate-4-phosphatase type II B
10423	CDIPT	CDP-diacylglycerol--inositol 3-phosphatidyltransferase
91860	CALML4	calmodulin like 4
3705	ITPK1	inositol-tetrakisphosphate 1-kinase
5296	PIK3R2	phosphoinositide-3-kinase regulatory subunit 2
51447	IP6K2	inositol hexakisphosphate kinase 2
4534	MTM1	myotubularin 1
3632	INPP5A	inositol polyphosphate-5-phosphatase A
55300	PI4K2B	phosphatidylinositol 4-kinase type 2 beta
8395	PIP5K1B	phosphatidylinositol-4-phosphate 5-kinase type 1 beta
5298	PI4KB	phosphatidylinositol 4-kinase beta
5335	PLCG1	phospholipase C gamma 1
23262	PPIP5K2	diphosphoinositol pentakisphosphate kinase 2
8396	PIP4K2B	phosphatidylinositol-5-phosphate 4-kinase type 2 beta
55613	MTMR8	myotubularin related protein 8
3636	INPPL1	inositol polyphosphate phosphatase like 1
3633	INPP5B	inositol polyphosphate-5-phosphatase B
51196	PLCE1	phospholipase C epsilon 1
22908	SACM1L	SAC1 like phosphatidylinositide phosphatase
2538	G6PC	glucose-6-phosphatase catalytic subunit
2203	FBP1	fructose-bisphosphatase 1
5210	PFKFB4	6-phosphofructo-2-kinase/fructose-2,6-biphosphatase 4
3172	HNF4A	hepatocyte nuclear factor 4 alpha
5211	PFKL	phosphofructokinase, liver type
1938	EEF2	eukaryotic translation elongation factor 2
5207	PFKFB1	6-phosphofructo-2-kinase/fructose-2,6-biphosphatase 1
3643	INSR	insulin receptor
2997	GYS1	glycogen synthase 1
5523	PPP2R3A	protein phosphatase 2 regulatory subunit B''alpha
5106	PCK2	phosphoenolpyruvate carboxykinase 2, mitochondrial
5563	PRKAA2	protein kinase AMP-activated catalytic subunit alpha 2
5468	PPARG	peroxisome proliferator activated receptor gamma
2998	GYS2	glycogen synthase 2
5105	PCK1	phosphoenolpyruvate carboxykinase 1
10645	CAMKK2	calcium/calmodulin dependent protein kinase kinase 2
32	ACACB	acetyl-CoA carboxylase beta
2308	FOXO1	forkhead box O1
5562	PRKAA1	protein kinase AMP-activated catalytic subunit alpha 1
55437	STRADB	STE20-related kinase adaptor beta
5213	PFKM	phosphofructokinase, muscle

23216	TBC1D1	TBC1 domain family member 1
1080	CFTR	cystic fibrosis transmembrane conductance regulator
6319	SCD	stearoyl-CoA desaturase
79966	SCD5	stearoyl-CoA desaturase 5
11031	RAB31	RAB31, member RAS oncogene family
30846	EHD2	EH domain containing 2
1213	CLTC	clathrin heavy chain
163	AP2B1	adaptor related protein complex 2 subunit beta 1
10890	RAB10	RAB10, member RAS oncogene family
55738	ARFGAP1	ADP ribosylation factor GTPase activating protein 1
10095	ARPC1B	actin related protein 2/3 complex subunit 1B
79720	VPS37B	VPS37B, ESCRT-I subunit
2348	FOLR1	folate receptor 1
30844	EHD4	EH domain containing 4
23527	ACAP2	ArfGAP with coiled-coil, ankyrin repeat and PH domains 2
84313	VPS25	vacuolar protein sorting 25 homolog
11311	VPS45	vacuolar protein sorting 45 homolog
51100	SH3GLB1	SH3 domain containing GRB2 like, endophilin B1
10094	ARPC3	actin related protein 2/3 complex subunit 3
11267	SNF8	SNF8, ESCRT-II complex subunit
387680	WASHC2A	WASH complex subunit 2A
10552	ARPC1A	actin related protein 2/3 complex subunit 1A
137492	VPS37A	VPS37A, ESCRT-I subunit
8853	ASAP2	ArfGAP with SH3 domain, ankyrin repeat and PH domain 2
375	ARF1	ADP ribosylation factor 1
80223	RAB11FIP1	RAB11 family interacting protein 1
2352	FOLR3	folate receptor 3
8724	SNX3	sorting nexin 3
253725	WASHC2C	WASH complex subunit 2C
22905	EPN2	epsin 2
160	AP2A1	adaptor related protein complex 2 subunit alpha 1
9559	VPS26A	VPS26, retromer complex component A
81873	ARPC5L	actin related protein 2/3 complex subunit 5 like
55616	ASAP3	ArfGAP with SH3 domain, ankyrin repeat and PH domain 3
857	CAV1	caveolin 1
4218	RAB8A	RAB8A, member RAS oncogene family
116984	ARAP2	ArfGAP with RhoGAP domain, ankyrin repeat and PH domain 2
829	CAPZA1	capping actin protein of muscle Z-line subunit alpha 1
867	CBL	Cbl proto-oncogene
5371	PML	promyelocytic leukemia
1211	CLTA	clathrin light chain A
3800	KIF5C	kinesin family member 5C
9265	CYTH3	cytohesin 3
3949	LDLR	low density lipoprotein receptor
30845	EHD3	EH domain containing 3
10109	ARPC2	actin related protein 2/3 complex subunit 2
8411	EEA1	early endosome antigen 1
11021	RAB35	RAB35, member RAS oncogene family
23325	WASHC4	WASH complex subunit 4
56288	PARD3	par-3 family cell polarity regulator
6643	SNX2	sorting nexin 2

9146	HGS	hepatocyte growth factor-regulated tyrosine kinase substrate
858	CAV2	caveolin 2
5868	RAB5A	RAB5A, member RAS oncogene family
7314	UBB	ubiquitin B
3310	HSPA6	heat shock protein family A (Hsp70) member 6
50807	ASAP1	ArfGAP with SH3 domain, ankyrin repeat and PH domain 1
3799	KIF5B	kinesin family member 5B
58533	SNX6	sorting nexin 6
440073	IQSEC3	IQ motif and Sec7 domain 3
26286	ARFGAP3	ADP ribosylation factor GTPase activating protein 3
27243	CHMP2A	charged multivesicular body protein 2A
57132	CHMP1B	charged multivesicular body protein 1B
55040	EPN3	epsin 3
8729	GBF1	golgi brefeldin A resistant guanine nucleotide exchange factor 1
9829	DNAJC6	DnaJ heat shock protein family (Hsp40) member C6
5662	PSD	pleckstrin and Sec7 domain containing
7852	CXCR4	C-X-C motif chemokine receptor 4
30011	SH3KBP1	SH3 domain containing kinase binding protein 1
51534	VTA1	vesicle trafficking 1
7189	TRAF6	TNF receptor associated factor 6
89853	MVB12B	multivesicular body subunit 12B
51028	VPS36	vacuolar protein sorting 36 homolog
9267	CYTH1	cytohesin 1
10564	ARFGEF2	ADP ribosylation factor guanine nucleotide exchange factor 2
118813	ZFYVE27	zinc finger FYVE-type containing 27
23327	NEDD4L	neural precursor cell expressed, developmentally down-regulated 4-like, E3 ubiquitin protein ligase
838	CASP5	caspase 5
10628	TXNIP	thioredoxin interacting protein
3665	IRF7	interferon regulatory factor 7
7158	TP53BP1	tumor protein p53 binding protein 1
4940	OAS3	2'-5'-oligoadenylate synthetase 3
10910	SUGT1	SGT1 homolog, MIS12 kinetochore complex assembly cochaperone
4938	OAS1	2'-5'-oligoadenylate synthetase 1
7295	TXN	thioredoxin
114769	CARD16	caspase recruitment domain family member 16
4793	NFKBIB	NFKB inhibitor beta
8767	RIPK2	receptor interacting serine/threonine kinase 2
118429	ANTXR2	ANTXR cell adhesion molecule 2
3428	IFI16	interferon gamma inducible protein 16
10392	NOD1	nucleotide binding oligomerization domain containing 1
9641	IKBKE	inhibitor of nuclear factor kappa B kinase subunit epsilon
55054	ATG16L1	autophagy related 16 like 1
837	CASP4	caspase 4
4939	OAS2	2'-5'-oligoadenylate synthetase 2
6041	RNASEL	ribonuclease L
79184	BRCC3	BRCA1/BRCA2-containing complex subunit 3
440068	CARD17	caspase recruitment domain family member 17
3661	IRF3	interferon regulatory factor 3
9474	ATG5	autophagy related 5
24145	PANX1	pannexin 1

9636	ISG15	ISG15 ubiquitin-like modifier
79132	DHX58	DExH-box helicase 58
5300	PIN1	peptidylprolyl cis/trans isomerase, NIMA-interacting 1
64135	IFIH1	interferon induced with helicase C domain 1
9755	TBKBP1	TBK1 binding protein 1
7187	TRAF3	TNF receptor associated factor 3
23586	DDX58	DExD/H-box helicase 58
115004	CGAS	cyclic GMP-AMP synthase
5441	POLR2L	RNA polymerase II subunit L
661	POLR3D	RNA polymerase III subunit D
9533	POLR1C	RNA polymerase I and III subunit C
5440	POLR2K	RNA polymerase II subunit K
55703	POLR3B	RNA polymerase III subunit B
10622	POLR3G	RNA polymerase III subunit G
10621	POLR3F	RNA polymerase III subunit F
84265	POLR3GL	RNA polymerase III subunit G like
103	ADAR	adenosine deaminase, RNA specific
51728	POLR3K	RNA polymerase III subunit K
10623	POLR3C	RNA polymerase III subunit C
11128	POLR3A	RNA polymerase III subunit A
6280	S100A9	S100 calcium binding protein A9
8061	FOSL1	FOS like 1, AP-1 transcription factor subunit
23765	IL17RA	interleukin 17 receptor A
4322	MMP13	matrix metallopeptidase 13
2354	FOSB	FosB proto-oncogene, AP-1 transcription factor subunit
1994	ELAVL1	ELAV like RNA binding protein 1
7184	HSP90B1	heat shock protein 90 beta family member 1
1557	CYP2C19	cytochrome P450 family 2 subfamily C member 19
2330	FMO5	flavin containing monooxygenase 5
4129	MAOB	monoamine oxidase B
2329	FMO4	flavin containing monooxygenase 4
4128	MAOA	monoamine oxidase A
127	ADH4	alcohol dehydrogenase 4 (class II), pi polypeptide
7367	UGT2B17	UDP glucuronosyltransferase family 2 member B17
2938	GSTA1	glutathione S-transferase alpha 1
2948	GSTM4	glutathione S-transferase mu 4
79799	UGT2A3	UDP glucuronosyltransferase family 2 member A3
130	ADH6	alcohol dehydrogenase 6 (class V)
2326	FMO1	flavin containing monooxygenase 1
2941	GSTA4	glutathione S-transferase alpha 4
128	ADH5	alcohol dehydrogenase 5 (class III), chi polypeptide
2939	GSTA2	glutathione S-transferase alpha 2
51733	UPB1	beta-ureidopropionase 1
2990	GUSB	glucuronidase beta
10	NAT2	N-acetyltransferase 2
1066	CES1	carboxylesterase 1
1806	DPYD	dihydropyrimidine dehydrogenase
54963	UCKL1	uridine-cytidine kinase 1 like 1
8824	CES2	carboxylesterase 2
2946	GSTM2	glutathione S-transferase mu 2
83549	UCK1	uridine-cytidine kinase 1

978	CDA	cytidine deaminase
22	ABCB7	ATP binding cassette subfamily B member 7
1244	ABCC2	ATP binding cassette subfamily C member 2
23461	ABCA5	ATP binding cassette subfamily A member 5
368	ABCC6	ATP binding cassette subfamily C member 6
23456	ABCB10	ATP binding cassette subfamily B member 10
20	ABCA2	ATP binding cassette subfamily A member 2
19	ABCA1	ATP binding cassette subfamily A member 1
10060	ABCC9	ATP binding cassette subfamily C member 9
10058	ABCB6	ATP binding cassette subfamily B member 6 (Langereis blood group)
8714	ABCC3	ATP binding cassette subfamily C member 3
5244	ABCB4	ATP binding cassette subfamily B member 4
5826	ABCD4	ATP binding cassette subfamily D member 4
9619	ABCG1	ATP binding cassette subfamily G member 1
10349	ABCA10	ATP binding cassette subfamily A member 10
21	ABCA3	ATP binding cassette subfamily A member 3
6518	SLC2A5	solute carrier family 2 member 5
6523	SLC5A1	solute carrier family 5 member 1
776	CACNA1D	calcium voltage-gated channel subunit alpha1 D
2542	SLC37A4	solute carrier family 37 member 4
481	ATP1B1	ATPase Na+/K+ transporting subunit beta 1
6476	SI	sucrase-isomaltase
6514	SLC2A2	solute carrier family 2 member 2
93432	MGAM2	maltase-glucoamylase 2 (putative)
8972	MGAM	maltase-glucoamylase
476	ATP1A1	ATPase Na+/K+ transporting subunit alpha 1
7512	XPNPEP2	X-prolyl aminopeptidase 2
11136	SLC7A9	solute carrier family 7 member 9
117247	SLC16A10	solute carrier family 16 member 10
4224	MEP1A	meprin A subunit alpha
6519	SLC3A1	solute carrier family 3 member 1
206358	SLC36A1	solute carrier family 36 member 1
9056	SLC7A7	solute carrier family 7 member 7
6564	SLC15A1	solute carrier family 15 member 1
1803	DPP4	dipeptidyl peptidase 4
1277	COL1A1	collagen type I alpha 1 chain
1291	COL6A1	collagen type VI alpha 1 chain
6505	SLC1A1	solute carrier family 1 member 1
1358	CPA2	carboxypeptidase A2
59272	ACE2	angiotensin I converting enzyme 2
1303	COL12A1	collagen type XII alpha 1 chain
5547	PRCP	prolylcarboxypeptidase
54407	SLC38A2	solute carrier family 38 member 2
3783	KCNN4	potassium calcium-activated channel subfamily N member 4
1290	COL5A2	collagen type V alpha 2 chain
4547	MTTP	microsomal triglyceride transfer protein
8611	PLPP1	phospholipid phosphatase 1
2169	FABP2	fatty acid binding protein 2
335	APOA1	apolipoprotein A1
8694	DGAT1	diacylglycerol O-acyltransferase 1
80168	MOGAT2	monoacylglycerol O-acyltransferase 2

84647	PLA2G12B	phospholipase A2 group XIIB
338	APOB	apolipoprotein B
5408	PNLIPRP2	pancreatic lipase related protein 2 (gene/pseudogene)
346606	MOGAT3	monoacylglycerol O-acyltransferase 3
949	SCARB1	scavenger receptor class B member 1
39	ACAT2	acetyl-CoA acetyltransferase 2
84649	DGAT2	diacylglycerol O-acyltransferase 2
337	APOA4	apolipoprotein A4
1056	CEL	carboxyl ester lipase
9963	SLC23A1	solute carrier family 23 member 1
8029	CUBN	cubilin
6948	TCN2	transcobalamin 2
151056	PLB1	phospholipase B1
80704	SLC19A3	solute carrier family 19 member 3
686	BTD	biotinidase
55788	LMBRD1	LMBR1 domain containing 1
5948	RBP2	retinol binding protein 2

Supplementary Table S5. The names of the 483 genes that were visualized in Figure 6

ENTREZID	SYMBOL	GENENAME
248	ALPI	alkaline phosphatase, intestinal
1670	DEFA5	defensin alpha 5
1671	DEFA6	defensin alpha 6
4295	MLN	motilin
4225	MEP1B	meprin A subunit beta
338	APOB	apolipoprotein B
3938	LCT	lactase
6476	SI	sucrase-isomaltase
79853	TM4SF20	transmembrane 4 L six family member 20
64240	ABCG5	ATP binding cassette subfamily G member 5
2172	FABP6	fatty acid binding protein 6
5651	TMPRSS15	transmembrane serine protease 15
2695	GIP	gastric inhibitory polypeptide
5068	REG3A	regenerating family member 3 alpha
4224	MEP1A	meprin A subunit alpha
339	APOBEC1	apolipoprotein B mRNA editing enzyme catalytic subunit 1
4547	MTTP	microsomal triglyceride transfer protein
2169	FABP2	fatty acid binding protein 2
1576	CYP3A4	cytochrome P450 family 3 subfamily A member 4
795	S100G	S100 calcium binding protein G
54860	MS4A12	membrane spanning 4-domains A12
229	ALDOB	aldolase, fructose-bisphosphate B
343	AQP8	aquaporin 8
6527	SLC5A4	solute carrier family 5 member 4
10022	INSL5	insulin like 5
4640	MYO1A	myosin IA
9154	SLC28A1	solute carrier family 28 member 1
6370	CCL25	C-C motif chemokine ligand 25
10	NAT2	N-acetyltransferase 2
56667	MUC13	mucin 13, cell surface associated
4583	MUC2	mucin 2, oligomeric mucus/gel-forming
2984	GUCY2C	guanylate cyclase 2C
54827	NXPE4	neurexophilin and PC-esterase domain family member 4
2981	GUCA2B	guanylate cyclase activator 2B
27299	ADAMDEC1	ADAM like decysin 1
63924	CIDEC	cell death inducing DFFA like effector c
1557	CYP2C19	cytochrome P450 family 2 subfamily C member 19
54658	UGT1A1	UDP glucuronosyltransferase family 1 member A1
640	BLK	BLK proto-oncogene, Src family tyrosine kinase
79154	DHRS11	dehydrogenase/reductase 11
11148	HHLA2	HERV-H LTR-associating 2
2742	GLRA2	glycine receptor alpha 2
5009	OTC	ornithine carbamoyltransferase
5357	PLS1	plastin 1
8435	SOAT2	sterol O-acyltransferase 2
347853	TBX10	T-box 10
24146	CLDN15	claudin 15
80168	MOGAT2	monoacylglycerol O-acyltransferase 2
1045	CDX2	caudal type homeobox 2

10004	NAALADL1	N-acetylated alpha-linked acidic dipeptidase like 1
23563	CHST5	carbohydrate sulfotransferase 5
1800	DPEP1	dipeptidase 1
3174	HNF4G	hepatocyte nuclear factor 4 gamma
9058	SLC13A2	solute carrier family 13 member 2
6523	SLC5A1	solute carrier family 5 member 1
1015	CDH17	cadherin 17
346562	GNAT3	G protein subunit alpha transducin 3
80022	MYO15B	myosin XVB
2494	NR5A2	nuclear receptor subfamily 5 group A member 2
79861	TUBAL3	tubulin alpha like 3
79908	BTNL8	butyrophilin like 8
8694	DGAT1	diacylglycerol O-acyltransferase 1
3960	LGALS4	galectin 4
64922	LRRC19	leucine rich repeat containing 19
5697	PYY	peptide YY
10050	SLC17A4	solute carrier family 17 member 4
55630	SLC39A4	solute carrier family 39 member 4
9032	TM4SF5	transmembrane 4 L six family member 5
7512	XPNPEP2	X-prolyl aminopeptidase 2
53630	BCO1	beta-carotene oxygenase 1
63928	CHP2	calcineurin like EF-hand protein 2
2938	GSTA1	glutathione S-transferase alpha 1
10481	HOXB13	homeobox B13
3957	LGALS2	galectin 2
6564	SLC15A1	solute carrier family 15 member 1
6514	SLC2A2	solute carrier family 2 member 2
6689	SPIB	Spi-B transcription factor
51066	SSUH2	ssu-2 homolog (C. elegans)
11181	TREH	trehalase
7366	UGT2B15	UDP glucuronosyltransferase family 2 member B15
29974	A1CF	APOBEC1 complementation factor
838	CASP5	caspase 5
973	CD79A	CD79a molecule
54825	CDHR2	cadherin related family member 2
53841	CDHR5	cadherin related family member 5
27141	CIDEB	cell death inducing DFFA like effector b
8529	CYP4F2	cytochrome P450 family 4 subfamily F member 2
1644	DDC	dopa decarboxylase
2168	FABP1	fatty acid binding protein 1
79368	FCRL2	Fc receptor like 2
3360	HTR4	5-hydroxytryptamine receptor 4
8856	NR1I2	nuclear receptor subfamily 1 group I member 2
5030	P2RY4	pyrimidinergic receptor P2Y4
79799	UGT2A3	UDP glucuronosyltransferase family 2 member A3
95	ACY1	aminoacylase 1
337	APOA4	apolipoprotein A4
80117	ARL14	ADP ribosylation factor like GTPase 14
8708	B3GALT1	beta-1,3-galactosyltransferase 1
55024	BANK1	B cell scaffold protein with ankyrin repeats 1
759	CA1	carbonic anhydrase 1

885	CCK	cholecystokinin
930	CD19	CD19 molecule
4166	CHST6	carbohydrate sulfotransferase 6
1179	CLCA1	chloride channel accessory 1
22802	CLCA4	chloride channel accessory 4
1755	DMBT1	deleted in malignant brain tumors 1
9514	GAL3ST1	galactose-3-O-sulfotransferase 1
26290	GALNT8	polypeptide N-acetylgalactosaminyltransferase 8
8811	GALR2	galanin receptor 2
10223	GPA33	glycoprotein A33
3769	KCNJ13	potassium voltage-gated channel subfamily J member 13
3795	KHK	ketohexokinase
54474	KRT20	keratin 20
10637	LEFTY1	left-right determination factor 1
126353	MISP	mitotic spindle positioning
931	MS4A1	membrane spanning 4-domains A1
4648	MYO7B	myosin VIIB
6344	SCTR	secretin receptor
27284	SULT1B1	sulfotransferase family 1B member 1
7104	TM4SF4	transmembrane 4 L six family member 4
79838	TMC5	transmembrane channel like 5
7166	TPH1	tryptophan hydroxylase 1
7429	VIL1	villin 1
29802	VPREB3	V-set pre-B cell surrogate light chain 3
653808	ZG16	zymogen granule protein 16
1636	ACE	angiotensin I converting enzyme
59272	ACE2	angiotensin I converting enzyme 2
51703	ACSL5	acyl-CoA synthetase long chain family member 5
100	ADA	adenosine deaminase
81693	AMN	amnion associated transmembrane protein
26	AOC1	amine oxidase, copper containing 1
23526	ARHGAP45	Rho GTPase activating protein 45
398	ARHGDIG	Rho GDP dissociation inhibitor gamma
474	ATOH1	atonal bHLH transcription factor 1
80115	BAIAP2L2	BAI1 associated protein 2 like 2
10917	BTNL3	butyrophilin like 3
766	CA7	carbonic anhydrase 7
1235	CCR6	C-C motif chemokine receptor 6
959	CD40LG	CD40 ligand
8029	CUBN	cubilin
1577	CYP3A5	cytochrome P450 family 3 subfamily A member 5
1943	EFNA2	ephrin A2
79574	EPS8L3	EPS8 like 3
8857	FCGBP	Fc fragment of IgG binding protein
9214	FCMR	Fc fragment of IgM receptor
2859	GPR35	G protein-coupled receptor 35
3001	GZMA	granzyme A
8369	HIST1H4G	histone cluster 1 H4 family member g
80201	HKDC1	hexokinase domain containing 1
3219	HOXB9	homeobox B9
3575	IL7R	interleukin 7 receptor

3805	KIR2DL4	killer cell immunoglobulin like receptor, two Ig domains and long cytoplasmic tail 4
93432	MGAM2	maltase-glucoamylase 2 (putative)
4495	MT1G	metallothionein 1G
29881	NPC1L1	NPC1 like intracellular cholesterol transporter 1
4922	NTS	neurotensin
5106	PCK2	phosphoenolpyruvate carboxykinase 2, mitochondrial
5125	PCSK5	proprotein convertase subtilisin/kexin type 5
3651	PDX1	pancreatic and duodenal homeobox 1
5331	PLCB3	phospholipase C beta 3
54866	PPP1R14D	protein phosphatase 1 regulatory inhibitor subunit 14D
54546	RNF186	ring finger protein 186
23314	SATB2	SATB homeobox 2
6425	SFRP5	secreted frizzled related protein 5
10166	SLC25A15	solute carrier family 25 member 15
1811	SLC26A3	solute carrier family 26 member 3
9153	SLC28A2	solute carrier family 28 member 2
54716	SLC6A20	solute carrier family 6 member 20
6750	SST	somatostatin
53345	TM6SF2	transmembrane 6 superfamily member 2
8876	VNN1	vanin 1
126	ADH1C	alcohol dehydrogenase 1C (class I), gamma polypeptide
54831	BEST2	bestrophin 2
91860	CALML4	calmodulin like 4
92211	CDHR1	cadherin related family member 1
1044	CDX1	caudal type homeobox 1
8824	CES2	carboxylesterase 2
1412	CRYBA2	crystallin beta A2
2330	FMO5	flavin containing monooxygenase 5
2838	GPR15	G protein-coupled receptor 15
57476	GRAMD1B	GRAM domain containing 1B
81618	ITM2C	integral membrane protein 2C
3706	ITPKA	inositol-trisphosphate 3-kinase A
4065	LY75	lymphocyte antigen 75
4063	LY9	lymphocyte antigen 9
8972	MGAM	maltase-glucoamylase
5026	P2RX5	purinergic receptor P2X 5
5078	PAX4	paired box 4
5452	POU2F2	POU class 2 homeobox 2
100533178	PRORY	proline rich Y-linked
5794	PTPRH	protein tyrosine phosphatase, receptor type H
6343	SCT	secretin
6462	SHBG	sex hormone binding globulin
6555	SLC10A2	solute carrier family 10 member 2
55065	SLC52A1	solute carrier family 52 member 1
159963	SLC5A12	solute carrier family 5 member 12
11136	SLC7A9	solute carrier family 7 member 9
6664	SOX11	SRY-box 11
26228	STAP1	signal transducing adaptor family member 1
26230	TIAM2	T cell lymphoma invasion and metastasis 2
80008	TMEM156	transmembrane protein 156

89870	TRIM15	tripartite motif containing 15
7103	TSPAN8	tetraspanin 8
13	AADAC	arylacetamide deacetylase
9625	AATK	apoptosis associated tyrosine kinase
5243	ABCB1	ATP binding cassette subfamily B member 1
1244	ABCC2	ATP binding cassette subfamily C member 2
215	ABCD1	ATP binding cassette subfamily D member 1
9429	ABCG2	ATP binding cassette subfamily G member 2 (Junior blood group)
28	ABO	ABO, alpha 1-3-N-acetylgalactosaminyltransferase and alpha 1-3-galactosyltransferase
51554	ACKR4	atypical chemokine receptor 4
72	ACTG2	actin, gamma 2, smooth muscle, enteric
11033	ADAP1	ArfGAP with dual PH domains 1
84830	ADTRP	androgen dependent TFPI regulating protein
79814	AGMAT	agmatinase
23382	AHCYL2	adenosylhomocysteinase like 2
57016	AKR1B10	aldo-keto reductase family 1 member B10
8644	AKR1C3	aldo-keto reductase family 1 member C3
22977	AKR7A3	aldo-keto reductase family 7 member A3
290	ANPEP	alanyl aminopeptidase, membrane
312	ANXA13	annexin A13
27237	ARHGEF16	Rho guanine nucleotide exchange factor 16
23120	ATP10B	ATPase phospholipid transporting 10B (putative)
476	ATP1A1	ATPase Na+/K+ transporting subunit alpha 1
9914	ATP2C2	ATPase secretory pathway Ca2+ transporting 2
5205	ATP8B1	ATPase phospholipid transporting 8B1
10677	AVIL	advillin
10317	B3GALT5	beta-1,3-galactosyltransferase 5
10331	B3GNT3	UDP-GlcNAc:betaGal beta-1,3-N-acetylglucosaminyltransferase 3
8537	BCAS1	breast carcinoma amplified sequence 1
79370	BCL2L14	BCL2 like 14
330	BIRC3	baculoviral IAP repeat containing 3
651	BMP3	bone morphogenetic protein 3
656	BMP8B	bone morphogenetic protein 8b
339479	BRINP3	BMP/retinoic acid inducible neural specific 3
683	BST1	bone marrow stromal cell antigen 1
685	BTC	betacellulin
53838	C11orf24	chromosome 11 open reading frame 24
64073	C19orf33	chromosome 19 open reading frame 33
79762	C1orf115	chromosome 1 open reading frame 115
760	CA2	carbonic anhydrase 2
762	CA4	carbonic anhydrase 4
726	CAPN5	calpain 5
10753	CAPN9	calpain 9
29775	CARD10	caspase recruitment domain family member 10
843	CASP10	caspase 10
839	CASP6	caspase 6
840	CASP7	caspase 7
23624	CBLC	Cbl proto-oncogene C
873	CBR1	carbonyl reductase 1
80323	CCDC68	coiled-coil domain containing 68

6346	CCL1	C-C motif chemokine ligand 1
6356	CCL11	C-C motif chemokine ligand 11
6369	CCL24	C-C motif chemokine ligand 24
11126	CD160	CD160 molecule
57126	CD177	CD177 molecule
83461	CDCA3	cell division cycle associated 3
999	CDH1	cadherin 1
1030	CDKN2B	cyclin dependent kinase inhibitor 2B
1040	CDS1	CDP-diacylglycerol synthase 1
634	CEACAM1	carcinoembryonic antigen related cell adhesion molecule 1
1048	CEACAM5	carcinoembryonic antigen related cell adhesion molecule 5
1087	CEACAM7	carcinoembryonic antigen related cell adhesion molecule 7
23491	CES3	carboxylesterase 3
1124	CHN2	chimerin 2
1132	CHRM4	cholinergic receptor muscarinic 4
1139	CHRNA7	cholinergic receptor nicotinic alpha 7 subunit
10518	CIB2	calcium and integrin binding family member 2
1159	CKMT1B	creatine kinase, mitochondrial 1B
1181	CLCN2	chloride voltage-gated channel 2
1365	CLDN3	claudin 3
1366	CLDN7	claudin 7
53405	CLIC5	chloride intracellular channel 5
7464	CORO2A	coronin 2A
1510	CTSE	cathepsin E
1525	CXADR	CXADR, Ig-like cell adhesion molecule
1562	CYP2C18	cytochrome P450 family 2 subfamily C member 18
1573	CYP2J2	cytochrome P450 family 2 subfamily J member 2
54905	CYP2W1	cytochrome P450 family 2 subfamily W member 1
66002	CYP4F12	cytochrome P450 family 4 subfamily F member 12
1600	DAB1	DAB1, reelin adaptor protein
10170	DHRS9	dehydrogenase/reductase 9
56616	DIABLO	diablo IAP-binding mitochondrial protein
79962	DNAJC22	DnaJ heat shock protein family (Hsp40) member C22
1773	DNASE1	deoxyribonuclease 1
1803	DPP4	dipeptidyl peptidase 4
1829	DSG2	desmoglein 2
1907	EDN2	endothelin 2
1908	EDN3	endothelin 3
1999	ELF3	E74 like ETS transcription factor 3
80237	ELL3	elongation factor for RNA polymerase II 3
2028	ENPEP	glutamyl aminopeptidase
5169	ENPP3	ectonucleotide pyrophosphatase/phosphodiesterase 3
957	ENTPD5	ectonucleoside triphosphate diphosphohydrolase 5
57089	ENTPD7	ectonucleoside triphosphate diphosphohydrolase 7
4072	EPCAM	epithelial cell adhesion molecule
2048	EPHB2	EPH receptor B2
2053	EPHX2	epoxide hydrolase 2
64787	EPS8L2	EPS8 like 2
23474	ETHE1	ETHE1, persulfide dioxygenase
51513	ETV7	ETS variant 7
2150	F2RL1	F2R like trypsin receptor 1

54854	FAM83E	family with sequence similarity 83 member E
2195	FAT1	FAT atypical cadherin 1
54738	FEV	FEV, ETS transcription factor
2249	FGF4	fibroblast growth factor 4
2281	FKBP1B	FK506 binding protein 1B
55640	FLVCR2	feline leukemia virus subgroup C cellular receptor family member 2
2346	FOLH1	folate hydrolase 1
2306	FOXD2	forkhead box D2
2295	FOXF2	forkhead box F2
8928	FOXH1	forkhead box H1
2444	FRK	fyn related Src family tyrosine kinase
2524	FUT2	fucosyltransferase 2
2525	FUT3	fucosyltransferase 3 (Lewis blood group)
2528	FUT6	fucosyltransferase 6
5349	FXYD3	FXYD domain containing ion transport regulator 3
7855	FZD5	frizzled class receptor 5
2626	GATA4	GATA binding protein 4
2650	GCNT1	glucosaminyl (N-acetyl) transferase 1, core 2
2651	GCNT2	glucosaminyl (N-acetyl) transferase 2 (I blood group)
9245	GCNT3	glucosaminyl (N-acetyl) transferase 3, mucin type
51301	GCNT4	glucosaminyl (N-acetyl) transferase 4, core 2
54857	GDPD2	glycerophosphodiester phosphodiesterase domain containing 2
54810	GIPC2	GIPC PDZ domain containing family member 2
2710	GK	glycerol kinase
2767	GNA11	G protein subunit alpha 11
29909	GPR171	G protein-coupled receptor 171
9721	GPRIN2	G protein regulated inducer of neurite outgrowth 2
79774	GRTP1	growth hormone regulated TBC protein 1
55876	GSDMB	gasdermin B
2948	GSTM4	glutathione S-transferase mu 4
474381	H2AFB2	H2A histone family member B2
9421	HAND1	heart and neural crest derivatives expressed 1
9843	HEPH	hephaestin
6927	HNF1A	HNF1 homeobox A
3172	HNF4A	hepatocyte nuclear factor 4 alpha
3238	HOXD12	homeobox D12
3239	HOXD13	homeobox D13
54979	HRASLS2	HRAS like suppressor 2
3291	HSD11B2	hydroxysteroid 11-beta dehydrogenase 2
51170	HSD17B11	hydroxysteroid 17-beta dehydrogenase 11
3294	HSD17B2	hydroxysteroid 17-beta dehydrogenase 2
3352	HTR1D	5-hydroxytryptamine receptor 1D
3549	IHH	indian hedgehog
3558	IL2	interleukin 2
3574	IL7	interleukin 7
55765	INAVA	innate immunity activator
3682	ITGAE	integrin subunit alpha E
3512	JCHAIN	joining chain of multimeric IgA and IgM
81033	KCNH6	potassium voltage-gated channel subfamily H member 6
54207	KCNK10	potassium two pore domain channel subfamily K member 10
8645	KCNK5	potassium two pore domain channel subfamily K member 5

688	KLF5	Kruppel like factor 5
55554	KLK15	kallikrein related peptidase 15
3859	KRT12	keratin 12
3880	KRT19	keratin 19
3856	KRT8	keratin 8
3927	LASP1	LIM and SH3 protein 1
10162	LPCAT3	lysophosphatidylcholine acyltransferase 3
79782	LRRC31	leucine rich repeat containing 31
10586	MAB21L2	mab-21 like 2
7851	MALL	mal, T cell differentiation protein like
11184	MAP4K1	mitogen-activated protein kinase kinase kinase kinase 1
4248	MGAT3	mannosyl (beta-1,4-)-glycoprotein beta-1,4-N-acetylglucosaminyltransferas
11282	MGAT4B	alpha-1,3-mannosyl-glycoprotein 4-beta-N-acetylglucosaminyltransferase B
4311	MME	membrane metalloendopeptidase
4324	MMP15	matrix metallopeptidase 15
55034	MOCOS	molybdenum cofactor sulfurase
4486	MST1R	macrophage stimulating 1 receptor
4496	MT1H	metallothionein 1H
10903	MTMR11	myotubularin related protein 11
4543	MTNR1A	melatonin receptor 1A
4585	MUC4	mucin 4, cell surface associated
79784	MYH14	myosin heavy chain 14
9	NAT1	N-acetyltransferase 1
9436	NCR2	natural cytotoxicity triggering receptor 2
50674	NEUROG3	neurogenin 3
579	NKX3-2	NK3 homeobox 2
4843	NOS2	nitric oxide synthase 2
27035	NOX1	NADPH oxidase 1
5540	NPY4R	neuropeptide Y receptor Y4
8431	NR0B2	nuclear receptor subfamily 0 group B member 2
51559	NT5DC3	5'-nucleotidase domain containing 3
4923	NTSR1	neurotensin receptor 1
4942	OAT	ornithine aminotransferase
10562	OLFM4	olfactomedin 4
4975	OMP	olfactory marker protein
9480	ONECUT2	one cut homeobox 2
79646	PANK3	pantothenate kinase 3
64081	PBLD	phenazine biosynthesis like protein domain containing
23590	PDSS1	decaprenyl diphosphate synthase subunit 1
79849	PDZD3	PDZ domain containing 3
79955	PDZD7	PDZ domain containing 7
5174	PDZK1	PDZ domain containing 1
5184	PEPD	peptidase D
23035	PHLPP2	PH domain and leucine rich repeat protein phosphatase 2
5284	PIGR	polymeric immunoglobulin receptor
80235	PIGZ	phosphatidylinositol glycan anchor biosynthesis class Z
5313	PKLR	pyruvate kinase L/R
8399	PLA2G10	phospholipase A2 group X
51316	PLAC8	placenta specific 8
55200	PLEKHG6	pleckstrin homology and RhoGEF domain containing G6
79949	PLEKHS1	pleckstrin homology domain containing S1

79983	POF1B	POF1B, actin binding protein
5450	POU2AF1	POU class 2 associating factor 1
51012	PRELID3B	PRELI domain containing 3B
5593	PRKG2	protein kinase cGMP-dependent 2
5625	PRODH	proline dehydrogenase 1
79170	PRR15L	proline rich 15 like
79899	PRR5L	proline rich 5 like
5652	PRSS8	serine protease 8
5753	PTK6	protein tyrosine kinase 6
5800	PTPRO	protein tyrosine phosphatase, receptor type O
80223	RAB11FIP1	RAB11 family interacting protein 1
64284	RAB17	RAB17, member RAS oncogene family
54884	RETSAT	retinol saturase
79589	RNF128	ring finger protein 128, E3 ubiquitin protein ligase
6195	RPS6KA1	ribosomal protein S6 kinase A1
85477	SCIN	scinderin
6398	SECTM1	secreted and transmembrane 1
8991	SELENBP1	selenium binding protein 1
6414	SELENOP	selenoprotein P
57715	SEMA4G	semaphorin 4G
80031	SEMA6D	semaphorin 6D
22928	SEPHS2	selenophosphate synthetase 2
6570	SLC18A1	solute carrier family 18 member A1
6505	SLC1A1	solute carrier family 1 member 1
5002	SLC22A18	solute carrier family 22 member 18
5003	SLC22A18AS	solute carrier family 22 member 18 antisense
1836	SLC26A2	solute carrier family 26 member 2
6518	SLC2A5	solute carrier family 2 member 5
54020	SLC37A1	solute carrier family 37 member 1
6519	SLC3A1	solute carrier family 3 member 1
80736	SLC44A4	solute carrier family 44 member 4
113235	SLC46A1	solute carrier family 46 member 1
283537	SLC46A3	solute carrier family 46 member 3
9497	SLC4A7	solute carrier family 4 member 7
6532	SLC6A4	solute carrier family 6 member 4
9056	SLC7A7	solute carrier family 7 member 7
6549	SLC9A2	solute carrier family 9 member A2
6550	SLC9A3	solute carrier family 9 member A3
55512	SMPD3	sphingomyelin phosphodiesterase 3
27293	SMPDL3B	sphingomyelin phosphodiesterase acid like 3B
6525	SMTN	smoothelin
27290	SPINK4	serine peptidase inhibitor, Kazal type 4
6692	SPINT1	serine peptidase inhibitor, Kunitz type 1
6717	SRI	sorcin
6751	SSTR1	somatostatin receptor 1
6768	ST14	suppression of tumorigenicity 14
55620	STAP2	signal transducing adaptor family member 2
55359	STYK1	serine/threonine/tyrosine kinase 1
6799	SULT1A2	sulfotransferase family 1A member 2
6783	SULT1E1	sulfotransferase family 1E member 1
6865	TACR2	tachykinin receptor 2

51567	TDP2	tyrosyl-DNA phosphodiesterase 2
27134	TJP3	tight junction protein 3
26007	TKFC	triokinase and FMN cyclase
100289255	TMEM238L	transmembrane protein 238 like
55321	TMEM74B	transmembrane protein 74B
7113	TMPRSS2	transmembrane serine protease 2
8792	TNFRSF11A	TNF receptor superfamily member 11a
608	TNFRSF17	TNF receptor superfamily member 17
9966	TNFSF15	TNF superfamily member 15
9540	TP53I3	tumor protein p53 inducible protein 3
25823	TPSG1	tryptase gamma 1
10107	TRIM10	tripartite motif containing 10
11074	TRIM31	tripartite motif containing 31
8989	TRPA1	transient receptor potential cation channel subfamily A member 1
57113	TRPC7	transient receptor potential cation channel subfamily C member 7
140803	TRPM6	transient receptor potential cation channel subfamily M member 6
10103	TSPAN1	tetraspanin 1
55020	TTC38	tetratricopeptide repeat domain 38
7367	UGT2B17	UDP glucuronosyltransferase family 2 member B17
54346	UNC93A	unc-93 homolog A
10083	USH1C	USH1 protein network component harmonin
7421	VDR	vitamin D receptor
50853	VILL	villin like
7432	VIP	vasoactive intestinal peptide
54621	VSIG10	V-set and immunoglobulin domain containing 10
7498	XDH	xanthine dehydrogenase
7504	XK	X-linked Kx blood group
51043	ZBTB7B	zinc finger and BTB domain containing 7B
